# Enhancing the therapeutic efficacy of dexamethasone for oral ulcers through adhesive gelatin hydrogel-based nano-delivery system

**DOI:** 10.1016/j.mtbio.2026.103341

**Published:** 2026-06-10

**Authors:** Qianwei Tang, Yanlin Chen, Meng Liu, Xueping Ning, Quanzhi Chen, Shengbin He

**Affiliations:** a Guangxi Medical University, Nanning, Guangxi, 530021, PR China; b Key Laboratory of Longevity and Aging related Diseases of Chinese Ministry of Education, University Engineering Research Center of Advanced Technologies in Medical and Biological Intelligent Manufacturing, Guangxi Colleges and Universities Key Laboratory of Biological Molecular Medicine Research, School of Basic Medical Sciences, Guangxi Medical University, Nanning, Guangxi, 530021, PR China; c Guangxi Clinical Research Center for Craniofacial Deformity, Guangxi Medical University College of Stomatology, Guangxi Medical University, Nanning, Guangxi, 530021, PR China

**Keywords:** Oral ulcer, Dexamethasone, Gelatin hydrogel, Polylactic acid, Effective treatment

## Abstract

With excellent anti-inflammatory and immunomodulatory performances, dexamethasone (Dex) is widely used in the treatment of skin and mucosal ulcers. However, its therapeutic efficacy for oral ulcers is limited by the unique oral environment. To address this issue, we loaded dexamethasone into polylactic acid nanoparticles (PANs) and modified them with ligands to enhance their targeted delivery efficiency. These Dex-loaded PANs (Dex-PANs) were further incorporated into gelatin hydrogel to prolong the release of the drug with a single administration. A self-synthesized bisuccinimidyl-ester was used, for the first time, as the cross-linker to covalently adhere the gelatin hydrogel to the ulcer site, thereby resisting mechanical detachment or salivary erosion. Compared to the control group, the tensile strength of the cross-linked gelatin hydrogel adhesive on skin was increased by approximately 10-fold. Moreover, the gelatin hydrogel could maintain for over 5 days in saliva, during which it slowly degraded without detachment from the skin while releasing the Dex-loaded PANs. In vitro experiments demonstrated that the Dex-PANs incorporated in gelatin hydrogel exhibited no cytotoxicity and promoted the proliferation of gingival epithelial cells, accelerating ulcer healing. In vivo studies showed that the novel Dex formulation enabled rapid healing of oral ulcers with a single administration. This approach may contribute to advancements in medical adhesives and guide the creation of nanoparticle-based buccal delivery technologies.

## Highlights

• A bisuccinimidyl-ester was designed and synthesized as the novel cross-linker.
• The cross-linker locked gelatin hydrogel onto ulcer site, resisting salivary erosion.
• Dexamethasone was loaded into polylactic acid nanoparticles to prepare Dex-PANs.
• Dopamine modification enhanced the targeting ability of Dex-PANs to macrophages.
• A hydrogel-based nano-delivery system was developed for oral ulcers treatment.

## Graphical abstract

**Figure:**
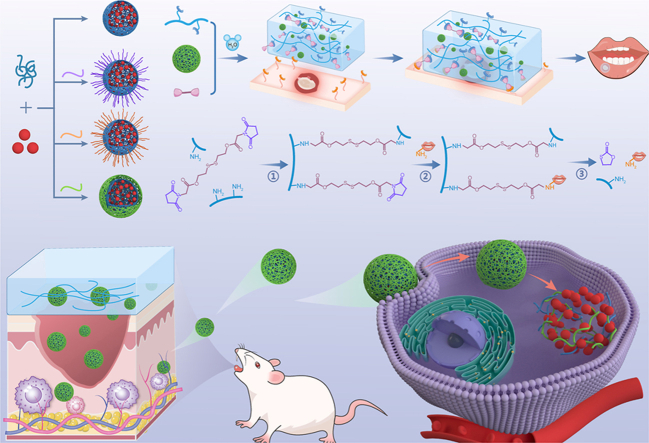

## 1 Introduction

As a prevalent oral condition, oral mucosal lesions (clinically termed oral ulcers) are characterized by continuous epithelial disruption or denudation in the buccal cavity. Epidemiological data indicate that approximately one-quarter of the worldwide adult population have been affected by this pathological condition at some point in their lifetime [[1], [2], [3]]. Once formed, ulcers may become secondarily infected, leading to local tissue inflammation and necrosis, thereby prolonging the disease course [[4], [5], [6]]. The accompanying pain and discomfort can exert significant negative impacts on patients' physiological and psychological states. Current pharmacological treatments for oral ulcers include glucocorticoids, tetracyclines, local anesthetics, antibacterial mouthwashes, and systemically administered immunomodulators [7,8]. Compared with systemic administration, locally applied glucocorticoid drugs are considered the most promising therapeutic approach, as they can effectively accelerate oral ulcer healing, alleviate pain, while avoiding the toxic side effects associated with systemic drug delivery [9,10].

Among the numerous glucocorticoid drugs, dexamethasone (Dex) demonstrates potent anti-inflammatory, immunosuppressive, and anti-allergic effects, and is widely used in the treatment of skin ulcers [11,12]. Typically, the Dex is mixed with polymers to prepare gels or patches for topical administration to skin ulcer sites. However, when these patches are applied to oral ulcers, their therapeutic efficacy is significantly compromised. This is because mechanical friction caused by physiological functions such as speech and chewing leads to the detachment of the patches, compounded by their erosion by saliva [[13], [14], [15]]. As a result, topical drug formulations struggle to remain on the affected areas of oral ulcers for sufficient durations, ultimately diminishing the localized drug enrichment effect and therapeutic efficacy. Furthermore, in the context of treating oral ulcers through anti-inflammatory and immunomodulatory therapy, the absorption efficiency of free Dex by target cells (macrophages) is notably low, further limiting the therapeutic effectiveness of conventional Dex administration [[16], [17], [18]].

Recently, the development of adhesive hydrogels represents a promising drug carrier for oral ulcer treatment. Adhesion mechanisms in these hydrogels are primarily classified as chemical or physical, with most systems relying on liquid-to-solid transition for physical attachment [[19], [20], [21], [22]]. The physical adhesion typically involves hydrogen bonding; yet, achieving robust adhesion remains difficult in the humid oral cavity due to environmental challenges. In contrast, chemical bonding strategies enable stronger tissue attachment while accommodating cellular mobility at the interface. However, currently available hydrogel patches prepared from polymers such as chitin, hyaluronic acid, and cellulose exhibit an average adhesion duration of less than 12 h to oral mucosa, necessitating repeated administration during treatment cycles [[23], [24], [25], [26]]. A critical unresolved challenge lies in the development of hydrogels with robust adhesive properties to reduce dosing frequency and improve patient experience.

Gelatin, a protein derived from the partial hydrolysis of collagen, has attracted significant attention due to its widespread availability, biocompatibility, edibility, and biodegradable nature [27,28]. These properties endow gelatin-based materials with substantial potential for applications in food packaging, biomedical fields including tissue engineering and wound healing, as well as pharmaceutical delivery systems [29,30]. Unlike collagen, gelatin offers cost-effectiveness and lacks immunogenicity under physiological conditions. Additionally, gelatin contains numerous integrin-binding sites that facilitate cellular adhesion and proliferation, making it particularly valuable for cell culture methodologies and regenerative medicine applications [31,32].

**Fig. 1:**
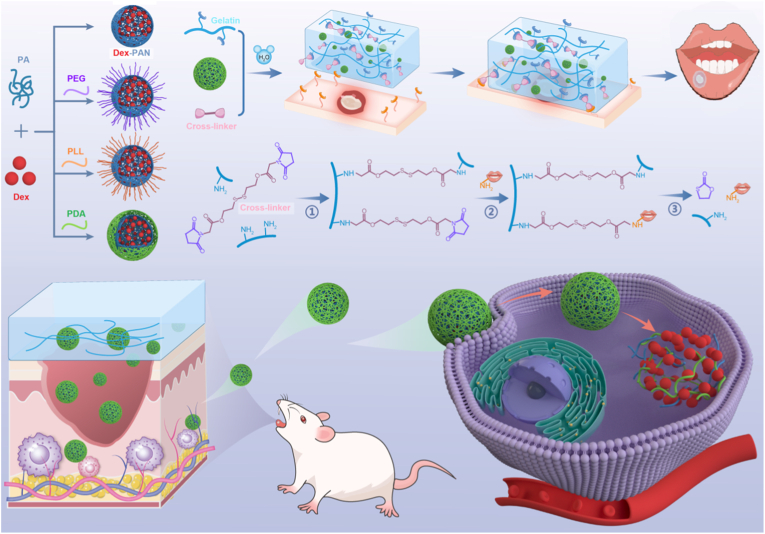
Schematic diagram of the adhesive gelatin hydrogel-based nano-delivery system.

In this study, a bisuccinimidyl-ester was designed and used for the rapid gelling of gelatin solution to form stable hydrogel that resists to salivary erosion. Furthermore, the hydrogel could be firmly adhered to the ulcer tissue through covalent bond, thereby resisting mechanical detachment. The gelatin hydrogel adhering to the ulcer tissue can maintain for over 5 days in a salivary environment while undergoing gradual degradation and sustained release of the encapsulated drug. To address the challenge of poor absorption of free Dex by macrophage, we further encapsulated Dex into polylactic acid nanoparticles (PANs) and modified them with dopamine (DPA) ligand, as shown in Fig. 1. These Dex-loaded PANs (Dex-PANs) were incorporated into the gelatin hydrogel to prolong the release of the nano-drug. Both in vitro and in vivo experiments demonstrated that the novel Dex formulation not only exhibited no toxicity but also promoted the anti-inflammatory of the ulcer and proliferation of gingival epithelial cells, accelerating the oral ulcer healing with a single administration.

## 2 Experimental section

### 2.1 Materials

The following reagents were obtained from the specified suppliers: dexamethasone (Dex), rhodamine (RhB) and dopamine (PDA) from Shanghai Yuanye Biological Technology Co., Ltd. (Shanghai, China); gelatin (Type B), poly-L-lysine (PLL), and polyethylene glycol (PEG6000) from Aladdin Bio-Chem Technology Co., Ltd. (Shanghai, China); glycine from Shanghai Sangon (Shanghai, China); polylactic acid (Mw 18-24 kDa), 4′,6-Diamidino-2-phenylindole (DAPI), phosgene, 2-hydroxyethyl disulfide, THF, NHS ester, and triethylamine from Sigma-Aldrich (St. Louis, USA). Rat IL-β ELISA Kit (JL20884), Rat TNF-α ELISA Kit (JL13202), and Rat lL-6 ELISA Kit (JL20896) were brought from JONLNBIO (Shanghai, China). Cell culture materials, including Dulbecco's Modified Eagle Medium (DMEM), were sourced from Gibco (Gaithersburg, MD, USA) and utilized directly. The cross-linker bisuccinimidyl-ester was synthesized by our laboratory as described in **Supplementary Materials Protocol S1**. Artificial saliva was bought from Merck (Darmstadt, Germany). The All additional solvents employed were of analytical grade quality.

### 2.2 Preparation of gelatin hydrogel and biological adhesion test

Type B gelatin of 10 mg was dissolved in 90 μL deionized water in a centrifuge tube. The tube was incubated at 70 °C for 10 min and cooled naturally to room temperature. A 10-μL aliquot of cross-linker solution (dissolved in DMSO) was added into the tube to achieve a final cross-linker concentration of 1-20 mg/mL. After mixed thoroughly, the solution was placed upright for 3 min before inverting to observe gelation. For the evaluation of adhesion and saliva resistance, fresh porcine skin was prepared by removing subcutaneous fat to expose the mucosal layer. The mixed gelatin/cross-linker solution (100 μL) was applied onto the mucosal surface to form a gelatin hydrogel patche with an area of ∼1 cm2‌ After 3 min’ gelation and adhesion, the skin with hydrogel patche were immersed in artificial saliva (37 °C, 200 rpm) for several days, renewing the saliva daily.

For physical adhesion, the mixed gelatin/cross-linker solution was applied onto a plastic film to form a gelatin hydrogel patche with an area of ∼1 cm2‌ After 3 min’ gelation, the hydrogel patche was detached from the plastic film and immersed in glycine solution (10 mg/mL) 3 min to terminated the cross-linking reaction. Then, the hydrogel patche was adhere physically onto the porcine skin and subjected to saliva resistance evaluation as described above.

### 2.3 Chemical analyses of the cross-linked gelatin hydrogel

The chemical structure of cross-linked gelatin was characterized using fourier transform infrared (FTIR) spectroscopy with a Thermo Scientific™ Nicolet™ iS10 spectrometer. Spectral data were acquired over the wavenumber range of 4000–400 cm^−1^, employing 32 co-added scans at a spectral resolution of 1 cm^−1^, with automated gain optimization to ensure signal consistency. Protein molecular weight distribution was assessed via sodium dodecyl sulfate–polyacrylamide gel electrophoresis (SDS-PAGE). A two-layer polyacrylamide gel system was employed, comprising a 5% (w/v) stacking gel and a 6% (w/v) resolving gel. Gelatin hydrogel were mechanically disrupted using fine-tipped forceps and solubilized in a 2× concentrated loading buffer formulated as follows: 1.2 mL distilled water, 1 mL glycerol, 1 mL of 20% (w/v) SDS, 0.25 mL of 1% (w/v) bromophenol blue, 0.075 g dithiothreitol (DTT), and 1 mL of 0.5 M Tris-HCl buffer (pH 6.8). Electrophoresis was conducted under standard conditions, followed by protein visualization through staining with 0.04% (w/v) Coomassie Brilliant Blue G-250 in a solution of 20% (v/v) methanol and 3.5% (v/v) perchloric acid. After a 2-h staining period, gels underwent three sequential destaining steps in 5% (v/v) acetic acid and 20% (v/v) methanol, each lasting 30 min. Molecular weight estimation of gelatin polypeptides was performed by comparison with a pre-stained protein ladder spanning 10 to 180 kDa.

### 2.4 Characterization of the adhesive gelatin hydrogel

The ex vivo adhesion of the gelatin hydrogel on mucosal tissue was assessed by the following method. Fresh porcine skin was degreased to expose the mucosal layer. A gelatin/cross-linker mixture of 100 μL, containing 0.1 g/mL gelatin and cross-linker of different concentrations, was applied between two layers of the exposed mucosal tissues, and the two layers were compressed for 3 min to facilitate cross-linking between the gelatin hydrogel and mucosal tissues. The tensile properties of the bonded joints were evaluated via an Instron 5567 mechanical testing system at a crosshead speed of 10 mm/min. For in vivo mucoadhesion assessment, 6-week-old SD rats weighing 200 ± 10 g were utilized. Following overnight fasting with ad libitum water access, animals were anesthetized using 1% pentobarbital sodium (40 mg/kg). Subsequently, gelatin/cross-linker mixtures of 100 μL, with 0.1 g/mL gelatin and cross-linker of different concentrations, were administered onto the buccal mucosa. After a 12-h administration period, the adherence condition of the gelatin hydrogel to the buccal tissue was evaluated and documented. For the electron microscope observation of the hydrogel, a cross-linked gelatin hydrogel was prepared using a similar method as described above. After freeze-drying, the surface and cross-section (under liquid nitrogen-induced cryo-cracking) of the gel were observed using a scanning electron microscope (FE-SEM SU8020, Japan).

### 2.5 Preparation and characterization of modified polylactic acid nanoparticles

The synthesis of polylactic acid nanoparticles (PANs) was conducted via a solvent displacement method, with distinct surface modifications achieved through controlled polymer conjugation. Polylactic acid of 20 mg was dissolved in 1 mL of acetonitrile, and the solution was introduced into 20 mL polyethylene glycol (PEG) aqueous solution (1%, w/v). The mixture was ultrasonicated for 4 h, yielding PEGylated nanoparticles (PEG-PANs). The resulting colloidal suspension was isolated by centrifugation at 12,000 × g for 20 min, followed by three sequential washes with distilled water and subsequent redispersion in ultrapure H_2_O. To generate poly-L-lysine-functionalized variants (PLL-PANs), the PEG component was substituted with 4% (w/v) poly-L-lysine under identical process parameters. Unmodified PANs were prepared in parallel without any surface modifier. For polydopamine (PDA) coating, freshly prepared PAN suspensions were exposed to an alkaline dopamine hydrochloride solution (0.5 mg/mL, pH 10, buffered with Tris-HCl) and incubated for 6 h under mild magnetic agitation, enabling spontaneous oxidative polymerization and conformal coating. The PDA-coated nanoparticles (PDA-PANs) were purified through three rounds of centrifugation at 10,000 × g for 30 min to remove unreacted monomers and oligomers.

Fluorescent (Rhodamine B-loaded) or pharmacologically active (dexamethasone-loaded) nanoparticle formulations were synthesized by co-dissolving the respective cargo (RhB or Dex) in the initial acetonitrile phase prior to nanoprecipitation, ensuring homogeneous drug encapsulation. All nanoparticle systems were comprehensively characterized using scanning electron microscopy (SEM) for morphology, UV-Vis spectroscopy for optical properties, fourier-transform infrared spectroscopy (FTIR) for chemical bonding analysis, fluorescence spectroscopy for dye emission profiling, and dynamic light scattering (DLS) via a Zetasizer Nano ZS90 for hydrodynamic size and zeta potential determination.

### 2.6 Targeted delivery assays

To assess the cellular internalization of RhB across distinct nanocarrier formulations, both macrophage RAW264.7 (4 × 10^5^ cells) and human gingival epithelial cells (HGEC, 4 × 10^5^ cells) were seeded in 6-well plates and cultured at 37 °C for 48 h. Following removal of the culture medium via triple rinsing with phosphate-buffered saline (PBS), fresh medium supplemented with RhB of distinct nanocarrier formulations (all at a fixed RhB concentration of 10 μg/mL) was introduced, and cells were incubated for an additional 4 h. Post-incubation, the drug-containing medium was aspirated and the monolayer was triplicated washed with PBS. Cells were then detached using trypsin-EDTA, collected by centrifugation at 1000 × g for 5 min, and subjected to three subsequent PBS washes before resuspension in PBS for downstream analyses. Nuclei were counterstained with 100 μL of DAPI (1 μg/mL) for 10 min, then rinsed thrice with PBS. For flow cytometry (FCM), resuspended cells were analyzed immediately using an Accuri C5 flow cytometer (BD, USA) with excitation at 355 nm (50 mW) and 488 nm (50 mW), and emission detection at 450 ± 20 nm (335 V for detection voltage) and 595 ± 20 nm (468 V for detection voltage) to quantify fluorescence for DAPI and RhB, respectively. For fluorescence microscopy, cells grown on coverslips were fixed with 200 μL of 4% paraformaldehyde for 10 min at room temperature, followed by three PBS washes. Fluorescence images were acquired using a Leica DMi8 inverted microscope to capture blue fluorescence (DAPI) and red fluorescence (RhB) simultaneously.

### 2.7 In vitro cytotoxicity evaluation

HGECs were seeded in 96-well plates at 1 × 10^5^ cells/well and cultured under 37 °C/5% CO_2_. Upon reaching confluent monolayers, gelatin hydrogel and PAN complexes were added to achieve final concentrations of 0, 1, 2, 5, 10, 20, 50, and 100 mg/mL across experimental groups. At 24, 48, and 72 h post-treatment, the medium was discarded and cells were triple-rinsed with PBS. At each timepoint, 10 μL of CCK-8 reagent was added per well, followed by 1-h incubation (37 °C/5% CO_2_). Absorbance (OD) was measured at 450 nm using a microplate reader, and cell viability percentages were calculated. For apoptosis assay, the HGECs were trypsinized and harvested, followed by two washes with ice-cold PBS via centrifugation (300 × g, 5 min) to remove residual serum. After supernatant removal, experimental samples were resuspended in 100 μL Annexin binding buffer, supplemented with 5 μL Annexin V-FITC, and incubated for 5 min at room temperature in the dark. Subsequently, 400 μL binding buffer and 10 μL PI staining solution were added, and samples were further incubated for 20 min under dark conditions. Untreated cells served as unstained controls. All samples were subjected to FCM analysis. The fluorescence detection used FITC (ex: 488 nm/em: 520 ± 30 nm) and PI (ex: 488 nm/em: 610 ± 15 nm) channels. Voltage settings for FSC, SSC, and fluorescence channels were adjusted using the unstained control. Compensation was calibrated with single-stained controls (FITC and PI) and a double-stained sample. Experimental samples were then analyzed to quantify apoptosis. Apoptotic cell percentages were calculated using FlowJo 7.6 software.

### 2.8 In vivo therapeutic efficacy evaluation

All animal experiments in this study were conducted in compliance with the guidelines of ICH-GCP and approved by the Animal Ethics Committee of Guangxi Medical University (Approval No.:202502017). Eighteen 6 week-old male SD rats were acclimatized in the laboratory for 3 days. Anesthesia was induced via intraperitoneal injection of 2% pentobarbital sodium (30 mg/kg). The buccal mucosa of the lower incisors was dried with sterile cotton balls and isolated with sterile cotton for moisture control. A circular filter paper disc (5 mm diameter) was immersed in 50% glacial acetic acid for 5 s, then immediately applied to the dried gingival mucosa for 60 s. Residual acetic acid was removed by gently wiping the area with sterile saline-soaked cotton balls, creating a 5-mm diameter oral mucosal ulcer. At 48 h post-ulcer induction, the rats were randomly divided into 6 groups (n = 6). Given that gender has no effect on oral ulcers, gender differences are not considered within the scope of this study. The control group received no treatment. Experimental groups were treated with PDA-Dex-PAN loaded gelatin patches (PDA-Dex-PAN loaded gelatin group) on day 1; gentle pressure was applied for 3 min to ensure adhesion. The gelatin patch was prepared by mixing 10% gelatin, 1% cross-linker, and 0.5 mg/mL PDA-Dex-PAN (containing 26% Dex) in 200 μL pure water. The patches were prepared and immediately applied to the ulcer. To elucidate the therapeutic effects of each component, we additionally established the following four groups: 1) treatment with blank gelatin patches (Gelatin group); 2) one-time topical application of free dexamethasone (Free Dex group); 3) administration of dexamethasone-loaded gelatin patches (Dex loaded gelatin group); 4) administration of PDA-PAN-loaded gelatin patches (PDA-PAN-loaded gelatin group). For free Dex group, 200 μL Dex solution (0.13 mg/mL) was spread on the ulcer.

Ulcer progression was monitored daily and photographed. At regular intervals, 20 μL of blood were collected, and the Dex concentration in the serum was analyzed by liquid chromatography with Kromasil C_18_. On day 5, 200 μL of blood was collected via venous puncture, and serum was separated by centrifugation. TNF-α, IL-6, and IL-1β concentrations were quantified by ELISA. On day 7, rats were sacrificed. Ulcerated oral mucosal tissues were harvested, fixed in 4% paraformaldehyde, embedded in paraffin, and sectioned at 5 μm for H&E and Masson's staining to evaluate epithelial morphology and collagen deposition. Major organs (liver, spleen, lung, heart, kidney) underwent identical processing and H&E staining for histopathological analysis.

## 3 Results and discussion

### 3.1 Synthesis of the cross-linker and its effect on gelatin gelation

**Fig. 2:**
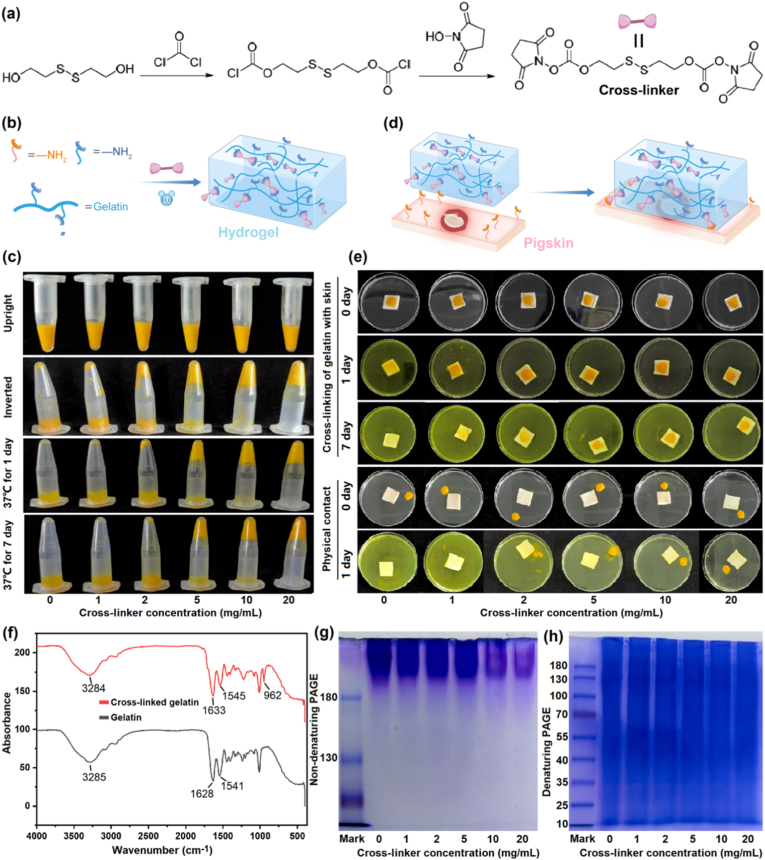
Preparation of the cross-linked gelatin hydrogel. (a) Synthesis procedure of the cross-linking agent. (b) Schematic illustration of the formation of cross-linked gelatin hydrogel. (c) Effect of cross-linker concentration on the gelation of gelatin solution. (d) Schematic diagram of the mechanism by which gelatin hydrogel adheres to skin. (e) Adhesion and water resistance of gelatin hydrogel on skin under artificial saliva conditions. (f) FTIR spectra of cross-linked gelatin. (g–h) Non-denaturing polyacrylamide gel electrophoresis (g) and denaturing electrophoresis (h) of the cross-linked gelatin. For (c) and (e), riboflavin (with yellow color) was loaded in the gelatin hydrogel to enhance its visibility. (For interpretation of the references to color in this figure legend, the reader is referred to the Web version of this article.)

To enable the rapid gelation of gelatin and its adhesion to mucosal tissue via covalent bonds, we designed and synthesized a symmetric bisuccinimidyl-ester cross-linker. This cross-linker can form amide bonds with amino groups on both gelatin and the mucosal surface through succinimide-activated ester chemistry, thereby achieving gelatin cross-linking and mucosal adhesion. Unlike conventional aldehyde-based cross-linkers that exhibited multiple toxicities [[33], [34], [35]], our cross-linker features two symmetric propionic acid N-hydroxysuccinimide esters connected by a disulfide bond. This design allows the cross-linker to degrade via a self-elimination reaction without producing toxic products [36,37], as shown in Fig. 1. Fig. 2 illustrates the synthesis route and schematic diagram of the cross-linker. NMR spectra confirmed the successful synthesis of the cross-linker, as shown in Supplementary Materials Fig. S1.

Given the rapid reaction kinetics between N-hydroxysuccinimide esters and amino groups [38,39], we hypothesized the bisuccinimidyl ester cross-linker would enable immediate gelation of gelatin solutions (Fig. 2b). As anticipated, gelatin underwent rapid cross-linking at cross-linker concentrations ≥5 mg/mL (Fig. 2c). The resulting hydrogel maintained structural integrity at physiological temperature (37 °C) for 7 days without dissolution, confirming enhanced thermal stability. To evaluate the mucoadhesion function, we applied the gelatin/cross-linker mixture onto mucosal tissue, enabling simultaneous gelation and covalent bonding to the tissue (Fig. 2d). Samples were then incubated in artificial saliva (37 °C, 7 days, agitation). Non-cross-linked or weakly cross-linked gels (<5 mg/mL cross-linker) dissolved rapidly (Fig. 2e), while mucoadhesive hydrogels cross-linked at ≥5 mg/mL retained stability for over 7 days. Although gradual hydrogel degradation occurred in saliva, no detachment from tissue was observed. Conversely, when residual active sites were blocked with glycine prior to application, physically adhered hydrogels detached immediately.

FTIR spectroscopy (Fig. 2f) was employed to characterize the cross-linked gelatin. Unmodified gelatin exhibited characteristic amide bond absorptions at 1628 cm^−1^ (amide I) and 1541 cm^−1^ (amide II) [28,40]. Upon cross-linking, both peaks underwent red-shifting, attributable to electron-withdrawing properties of the -O-CO-NH- moieties introduced by the cross-linker. A distinctive ester linkage (–CH_2_-O-CO-) emerged at ∼962 cm^−1^ in cross-linked gelatin, a spectral feature absent in native counterparts. These spectral alterations collectively verify successful covalent cross-linking of gelatin mediated by the symmetric bisuccinimidyl-ester cross-linker.

Fig. 2g and h presents native and denaturing polyacrylamide gel electrophoretic (PAGE) analyses of the cross-linked gelatin hydrogels, with different cross-linker concentrations. In native PAGE (Fig. 2g), higher cross-linker concentrations yielded progressively fainter gelatin bands, indicating that extensively cross-linked macromolecules failed to migrate into the resolving gel. Conversely, under denaturing conditions (Fig. 2h), all samples exhibited identical band patterns after cleavage of disulfide bonds within the cross-linker. This reversibility confirms that disulfide-mediated cross-linking enables gradual hydrogel degradation via glutathione reduction in physiological environments, facilitating sustained drug release.

### 3.2 Characterization of the adhesive gelatin hydrogel

**Fig. 3:**
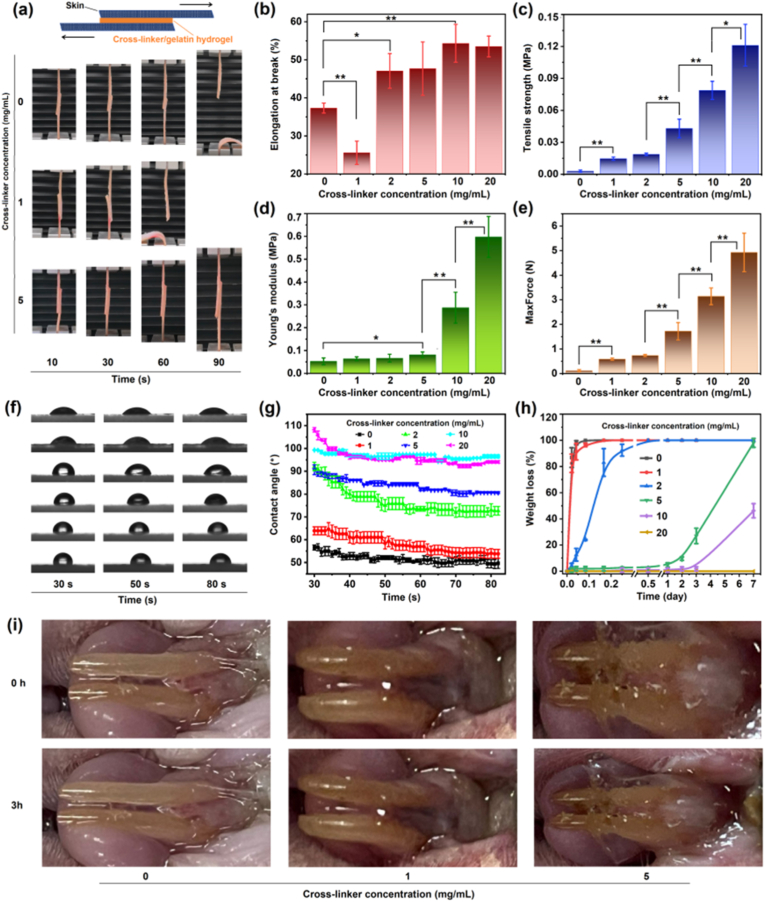
Characterization of the physical properties of the cross-linked gelatin hydrogel. (a) Tensile testing of the skins adhered with gelatin hydrogel. (b–e) Effect of cross-linker concentrations on elongation at break, tensile strength, Young's modulus, and maximum force. The data are represented as mean ± SD (n = 3). The difference analyses were performed using the Student's t-test. The difference was significant, as indicated by one asterisk for p < 0.05 and two asterisks for p < 0.01. (f–g) Influence of cross-linker concentrations on the contact angle of gelatin hydrogel. (h) Effect cross-linker concentrations on the erosion kinetics of gelatin hydrogel. (i) Influence of cross-linker concentrations on adhesion of gelatin hydrogel to rat oral mucosa.

The adhesion strength of gelatin hydrogels to mucosal skin was quantitatively evaluated by employing a cross-linker/gelatin hydrogel mixture as an adhesive to bond two pieces of porcine skin, as illustrated in Fig. 3a. Tensile tests were performed on the bonded samples using a universal testing machine. The tension resistance of the porcine skin bonded by the cross-linked gelatin hydrogel exhibited a positive correlation with cross-linker concentration, as shown in Fig. 3b–e. At a cross-linker concentration of 5 mg/mL, the elongation at break increased by 27 ± 16%, the tensile strength by 1333 ± 266%, the Young's modulus by 77 ± 23%, and the maximum force by 1343 ± 216% compared to the uncross-linked control. When the cross-linker concentration was increased to 20 mg/mL, these parameters further increased by 43 ± 7%, 4103 ± 453%, 1040 ± 145%, and 4034 ± 612%, respectively, demonstrating that the mechanical performance of the cross-linked gelatin hydrogel adhesive is significantly superior to that of the uncross-linked counterpart.

Fig. 3f and g presents the dynamic contact angles of cross-linked gelatin hydrogels. As the cross-linker concentration increased, the contact angle progressively rose, indicating enhanced hydrophobicity relative to the uncross-linked gelatin. This hydrophobic enhancement originates from the hydrophobic alkyl groups abundant in the cross-linker, which directly improves the hydrogel's resistance to aqueous erosion. Fig. 3h depicts the erosion dynamics of artificial saliva on gelatin hydrogels with varying degrees of cross-linking. The uncross-linked and 1 mg/mL cross-linker groups fully dissolved within 2 h. With increasing cross-linker concentration, the dissolution rate in saliva progressively decreased. At 10 mg/mL, the hydrogel exhibited less than 50% dissolution over 7 days; at 20 mg/mL, the hydrogel remained virtually intact, showing negligible degradation. Thus, the degradation rate of gelatin hydrogels in saliva can be precisely modulated by tuning the cross-linker concentration. Both the surface and interior of the cross-linked gelatin hydrogel possessed porous structures, with pore sizes mainly ranging from 2 to 8 μm (Fig. S2). These structures could serve as loading spaces for nanoparticles and release channels. Additionally, in vivo adhesion experiments demonstrated that the cross-linked gelatin hydrogel could firmly adhere to the oral mucosal surface (as shown in Fig. 3i), indicating its potential application in oral surface drug delivery.

### 3.3 Preparation and characterization of PANs of different modifications

Dexamethasone (Dex) exerts anti-inflammatory and wound-healing effects by modulating macrophage-mediated immune responses. However, the therapeutic efficacy of free Dex is severely limited by its low cellular uptake efficiency in macrophages. In contrast, cells can efficiently internalize drug-loaded nanoparticles via endocytosis and pinocytosis. Leveraging this principle, nanodrug delivery systems have been extensively explored for targeted cancer therapy [[41], [42], [43], [44]]. In this study, we developed a dexamethasone-loaded poly lactic acid nanoparticle (Dex-PAN) and functionalized its surface with specific ligands to enhance its targeted delivery to macrophages. To facilitate quantification of drug encapsulation efficiency and tracking of nanoparticle internalization, rhodamine B (RhB) was employed as a fluorescent surrogate for dexamethasone.

**Fig. 4:**
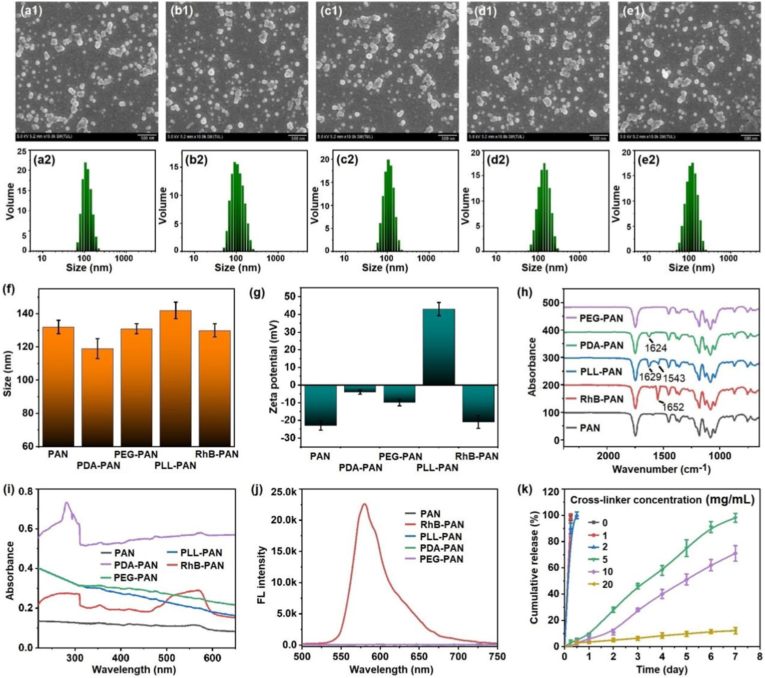
Preparation and characterization of polylactic acid nanoparticles. (a1–e1) SEM images of differently modified polylactic acid nanoparticles. (a2–e2) Hydrodynamic size distribution profiles of differently modified polylactic acid nanoparticles. (a) Polylactic acid nanoparticle (PAN). (b) PAN ‌functionalized with dopamine‌ (PDA-PAN). (c) PAN ‌functionalized with polyethylene glycol (PEG-PAN). (d) PAN ‌functionalized with poly-L-lysine (PLL-PAN). (e) PAN ‌loaded with rhodamine B (RhB-PAN). (f–g) Average hydrodynamic diameter (f) and Zeta potential (g) of differently modified polylactic acid nanoparticles. (h–j) FTIR spectra (h), UV-Vis absorption spectra (i), and fluorescence spectra (j) of differently modified polylactic acid nanoparticles. (k) Release kinetics of polylactic acid nanoparticles from the gelatin hydrogel. For (f), (g), and (k), the data are represented as mean ± SD (n = 3).

Fig. 4a1–e1, Fig. S3, and Fig. 4a2–e2 illustrate the size distribution of polylactic acid nanoparticles (PANs) with various ligand modifications. All formulations exhibited narrow and uniform size distributions, predominantly within the 50–200 nm range, with average size ranging from 106.4 ± 31.9 nm to 121.5 ± 34.5 nm (Fig. S3) and hydrodynamic diameters fluctuating between 119 ± 7 and 142 nm ± 5 (Fig. 4f). All samples met the fundamental size criterion (<500 nm) for effective nanodrug delivery [31,45]. Fig. 4g presents the zeta potential profiles of the PANs. Unmodified PANs exhibited a negative zeta potential due to ionization of surface carboxyl groups. Surface modification with dopamine (PDA, NH_3_^+^ ionization) and PEG (non-ionizable) increased the zeta potential, while polylysine (PLL) modification—due to its multiple primary amine groups—resulted in a distinct positive zeta potential. ‌Infrared spectroscopy‌ revealed that PANs ‌functionalized with PDA‌ (PDA-PAN) or ‌loaded with RhB (RhB-PAN)‌ exhibited a new absorption peak at ‌1624 cm^−1^‌ or ‌1543 cm^−1^‌, respectively, as shown in Fig. 4h. This peak is ‌attributed to‌ the ‌carbon-carbon double bonds‌ within the ‌aromatic ring of PDA or RhB‌. Upon ‌functionalization with PLL‌, the peaks corresponding to the ‌amide I band (1269 cm^−1^)‌ and ‌amide II band (1543 cm^−1^)‌ became particularly pronounced. ‌Fig. 4i‌ displays the ‌UV-Vis absorption spectra‌ of the PANs. Both PDA-PAN and RhB-PAN‌ exhibited an absorption peak ‌around 280 nm‌, indicating the ‌presence of a benzene ring structure‌. Additionally, RhB-PAN exhibited absorption ‌near 550 nm‌, ‌consistent with‌ the emission spectrum reported by Qiu and Li et al. [46,47].‌ The RhB-PAN emitted ‌intense fluorescence‌ in the ‌red spectral region (Fig. 4j)‌. These results collectively confirm the successful encapsulation of RhB within PANs and validate the effective surface functionalization with diverse ligands, establishing a robust platform for targeted macrophage delivery.

The RhB-PANs were incorporated into cross-linked gelatin hydrogels, and their release kinetics were evaluated in vitro. Artificial saliva supplemented with glutathione (GSH, 10 μM) was used to simulate the in vivo environment of the mucosal ulcer surface, allowing the translation of the in vitro release profile to an in vivo one. As shown in Fig. 4k, in hydrogels prepared with low cross-linker concentrations, the rapid release of RhB-PANs was observed. In contrast, hydrogels fabricated with higher cross-linker concentrations enabled sustained release of the nanoparticles, with release rates progressively decreasing as cross-linker concentration increased. At a cross-linker concentration of 20 mg/mL, negligible release was observed over the experimental period. Based on these findings, a cross-linker concentration of 10 mg/mL was selected for subsequent experiments, under which condition the RhB-PANs exhibited sustained release for over 7 days.

### 3.4 Targeted intracellular uptake of PANs with different modifications

**Fig. 5:**
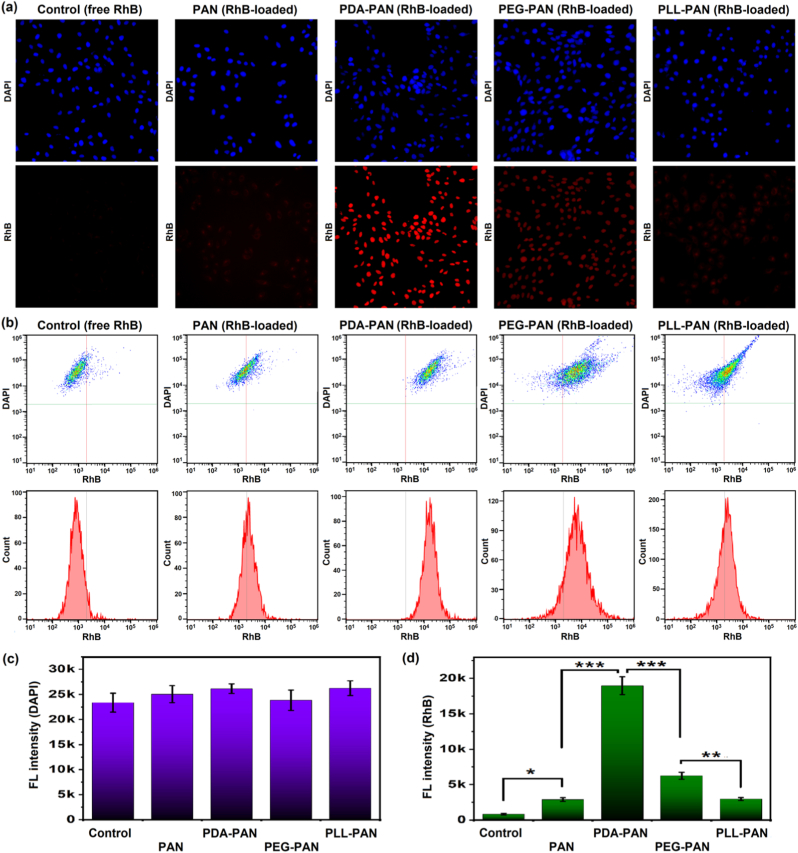
Uptake efficiency of differently modified polylactic acid nanoparticles by macrophages. (a) Observation of nanoparticle uptake by cells using fluorescence microscopy. (b) Flow cytometry analysis results of the aforementioned samples. (c–d) Statistical comparison of the corresponding mean fluorescence intensities of DAPI and RhB from FCM analysis. The data are represented as mean ± SD (n = 3). The difference analyses were performed using the Student's t-test, and the significance was indicated by one asterisk for p < 0.05, two asterisks for p < 0.01, and three asterisks for p < 0.001.

The limited targeting ability of free dexamethasone and its consequent off-target cytotoxicity necessitate a precision delivery strategy [48,49]. To address this, we evaluated the cell-specific uptake efficiency of ligand-modified PANs in macrophages and gingival epithelial cells (HGECs). Fig. 5a illustrates the fluorescent uptake of RhB-loaded PANs by macrophages. The weakest red fluorescence was observed in the free RhB group, confirming minimal cellular uptake of the unencapsulated drug. Encapsulation within PANs significantly enhanced uptake efficiency, and surface ligand modification profoundly influenced delivery performance. Quantitative flow cytometry (FCM) analysis (Fig. 5b–d) revealed the following ranking of macrophage uptake efficiency: ‌PDA-PAN > PEG-PAN > PLL-PAN =PAN > free RhB‌. In stark contrast, HGECs exhibited the highest uptake of ‌PLL-PAN, followed by PEG-PAN and ‌PDA-PAN, as shown in Fig. S4. Generally, the cellular uptake of nanoparticle occurs via the endocytic pathway, resulting in the entrapment of the nanoparticle in the endosome and eventually in the lysosome, where active degradation of polymer carrier and release of its content processes take place [50]. The endocytosis is governed by a confluence of factors—including particle surface chemistry, morphology, and cell-type-specific receptor expression profiles. Macrophages, rich in dopamine receptors, preferentially internalize PDA-functionalized nanoparticles via receptor-mediated endocytosis, a mechanism corroborated by prior reports from Mikusic and Qing et al. [51,52]. The addition of free PDA to compete for binding to cell surface receptors could inhibit the endocytosis of the PDA-PANs by macrophages, which verified that the endocytosis of PDA-PANs was mediated by PDA receptors on the macrophages (Fig. S5). Conversely, HGECs demonstrate a distinct receptor landscape favoring polylysine binding. This cell-type-dependent selectivity confirms that PDA-functionalized PAN achieve superior macrophage targeting while minimizing off-target accumulation in epithelial cells, thereby establishing a rational design principle for macrophage-specific dexamethasone delivery.

### 3.5 In vivo experiments demonstrated enhanced therapeutic efficacy of dexamethasone for oral ulcers

**Fig. 6:**
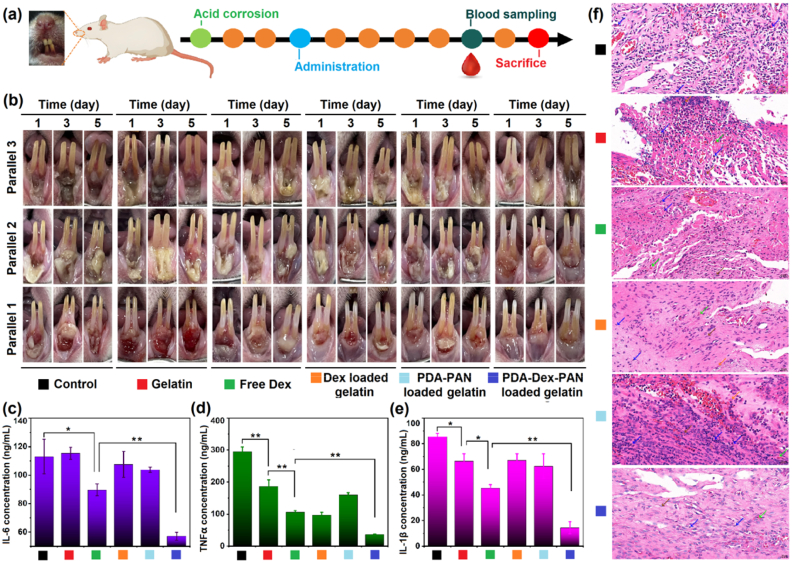
Therapeutic efficacy of gelatin hydrogel loaded with PDA-Dex-PAN on rat oral ulcers. (a) Schematic diagram of oral ulcer induction and drug administration in rat. (b) Therapeutic effects of different dexamethasone formulations on rat oral ulcers. A total of six replicates are present. The figure above shows three of them, and the other three replicates are provided in Figure S7 (c-e) Serum levels of inflammatory cytokines, including IL-6 (c), TNF-α (d), and IL-1β (e), in rats after treatment with different dexamethasone formulations. The data are represented as mean ± SD (n = 6). The difference analyses were performed using the Student's t-test, and the significance was indicated by one asterisk for p < 0.05 and two asterisks for p < 0.01. (f) H&E staining observation of the ulcer sites in rat oral mucosa after treatment with different dexamethasone formulations.

Oral mucosa, uniquely exposed to constant salivary flow and mechanical shear forces, presents a significant challenge for topical mucoadhesive drug delivery. Although recent studies have extended drug residence time in the oral cavity from minutes to hours using strongly adhesive hydrogels, persistent limitations—including low cellular drug uptake and inherently slow ulcer healing—still necessitate repeated dosing, compromising patient compliance [2,14,24]. To overcome these constraints, we leveraged the exceptional mucoadhesive properties of cross-linked gelatin hydrogels and the macrophage-targeting capability of Dex loaded PDA-PAN (PDA-Dex-PAN) to achieve curative outcomes with a single administration. As illustrated in Fig. 6a, a rat oral ulcer model was established via acid corrosion, followed by a single-dose application. Fig. S6a illustrates the serum pharmacokinetics of different Dex formulations. Free Dex administration exhibited rapid absorption and equally rapid metabolism, with complete clearance occurring within 24 h. Although the Dex loaded gelatin could prolong the serum retention time of Dex, this prolongation remained limited because of the large pore size of the gelatin network, which allows small-molecule drugs to be rapidly released from the gelatin gel. In contrast, PDA-Dex-PAN loaded gelatin significantly extended the serum retention time of Dex, which could be attributed to two factors: 1) the controlled release of PDA-Dex-PANs from the gelatin gel, and 2) the fact that PDA-Dex-PAN did not directly release Dex into the serum (as illustrated in Fig. S6b), but rather underwent cellular metabolism within target cells before being excreted into the serum. These results also demonstrated that the PDA-Dex-PAN loaded gelatin continuously released PDA-Dex-PANs following administration.

Fig. 6b and Fig. S7 exhibit the oral ulcer surface of rats under different treatment. Remarkably, rats treated with PDA-Dex-PAN loaded gelatin hydrogel exhibited complete ulcer healing within 5 days, whereas control groups and those receiving alternative formulations retained substantial wound areas. This demonstrates a dramatic acceleration in healing kinetics with the dual-function system: long-term release and efficient delivery. Notably, even the gelatin hydrogel group showed significantly enhanced ulcer repair compared to the untreated control, suggesting an intrinsic therapeutic contribution beyond mere drug delivery. This effect may arise from two synergistic mechanisms: (1) the highly cross-linked hydrogel acts as a physical barrier, isolating the wound from bacterial invasion and mechanical disruption; and (2) gelatin itself possesses bioactive properties that promote tissue regeneration and modulate inflammatory responses. Thus, the integrated platform not only enables sustained, targeted drug delivery but also actively supports the wound microenvironment, transforming a passive adhesive dressing into a therapeutically active system for one-time cure of oral ulcers.

To quantitatively evaluate the therapeutic effect of the PDA-Dex-PAN loaded gelatin hydrogel, the levels of interleukin IL-16, tumor necrosis factor TNF-α, and cytokine IL-1β in serum were measured using ELISA. The contents of these cytokines are positively correlated with the degree of inflammation and are widely used for assessing inflammatory status in mammals [18,48]. As shown in Fig. 6c–e, compared with the control group, both free Dex treatment and PDA-Dex-PAN loaded gelatin hydrogel treatment resulted in a decreasing trend in inflammatory factors. However, the PDA-Dex-PAN loaded gelatin hydrogel group exhibited the most significant reduction in inflammatory factors, further demonstrating that the Dex formulation prepared in this study, by loading into the gelatin hydrogel for slow release of targeted PDA-Dex-PAN, can substantially alleviate the inflammatory response of oral ulcers.

Fig. 6f presents the hematoxylin-eosin (HE) staining results of gingival tissues after 7 days of treatment. In the control group, no significant mucosal epithelium was observed in the gingival tissue, with small areas of necrosis and pyknotic or fragmented nuclei (orange arrows). There was extensive connective tissue hyperplasia in the lamina propria, with infiltration of numerous lymphocytes, granulocytes, and macrophages (blue arrows); slight hemorrhage was also noted (yellow curved polygon), indicating that the gingiva remained unrepaired and in an inflammatory state. Although treatment with gelatin or free dexamethasone led to some improvement, certain degrees of mucosal damage and inflammation persisted. In contrast, the gingival tissues of rats treated with PDA-Dex-PAN loaded gelatin hydrogel were primarily composed of stratified squamous epithelium and lamina propria, with intact mucosal epithelium. Occasionally, minute vacuoles were observed in the cytoplasm of mucosal epithelial cells (green curved polygon), along with localized hyperkeratosis (cyan arrows). Almost no cellular necrosis was detected; collagen fibers and fibroblasts were arranged in an interwoven pattern with neovascularization (green arrows), and only minimal lymphocyte infiltration (blue arrows) was observed, indicating that the ulcer was essentially repaired, and the inflammatory response had returned to normal levels. Masson staining revealed that, compared with the control group, the gingival tissues of rats treated with PDA-Dex-PAN loaded gelatin hydrogel exhibited extensive collagen fiber hyperplasia, as shown in Fig. S8. Together, these experimental results demonstrate that the combination of PDA-Dex-PANs with cross-linked gelatin hydrogel significantly enhances the therapeutic efficacy of Dex in treating oral ulcers in rats.

For clinically used oral ulcer adhesive patches, the patches are designed to dissolve rapidly in the moist oral environment, resulting in a short residence time on the lesion site. Consequently, they require multiple daily administrations to maintain therapeutic drug levels throughout the healing process. This repeated dosing regimen not only compromises patient compliance but also causes recurrent mechanical irritation and disruption of the healing mucosa. In contrast, our proposed system is engineered to provide prolonged mucosal adhesion and sustained drug release, enabling therapeutic equivalence with a single administration. This single-dose feature represents a significant practical advantage in terms of convenience, patient comfort, and adherence. Yet, the current results are based on an acute chemical ulcer model, which is a standard initial screening model but does not fully recapitulate chronic or immune-mediated oral ulcers.

### 3.6 Toxicity of the cross-linked gelatin hydrogel on gingival epithelial cells

**Fig. 7:**
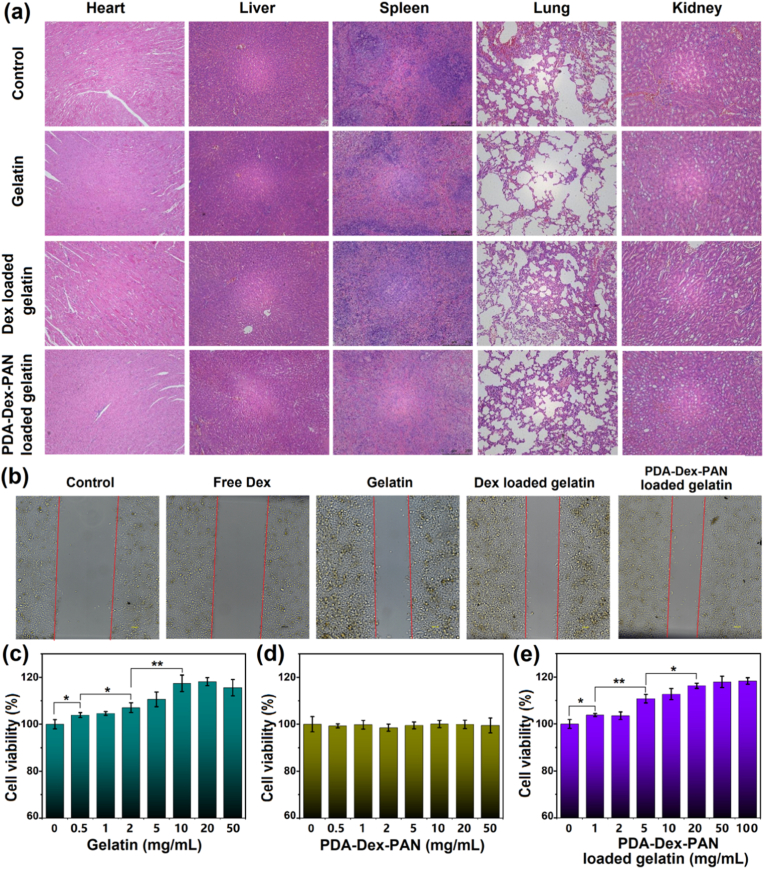
In vitro and in vivo toxicity experiments. (a) H&E staining of various rat organs following administration of Dex of different formulations. (b) Effect of gelatin hydrogel on the migration of gingival epithelial cells. (c-e) Effects of gelatin hydrogel (c), PDA-Dex-PAN (d), and PDA-Dex-PAN loaded gelatin hydrogel (e) on the proliferation of gingival epithelial cells. The data are represented as mean ± SD (n = 3). The difference analyses were performed using the Student's t-test, and the significance was indicated by one asterisk for p < 0.05 and two asterisks for p < 0.01.

The toxicity effects of the cross-linked gelatin hydrogel on major organs (heart, liver, spleen, lung, and kidney) in rats was assessed via HE staining. As shown in Fig. 7a, neither the plain gelatin hydrogel nor the hydrogels loaded with Dex or PDA-Dex-PAN exhibited signs of toxicity in these organs. No significant differences in nuclear density were observed across groups, indicating an absence of organ damage. Apoptosis assays confirmed that the both gelatin hydrogel and PDA-Dex-PAN loaded gelatin hydrogel, even at high concentrations (1–100 mg/mL), had no adverse effect on gingival epithelial cells and did not induce apoptosis (Fig. S9 and S10).

Further, the effect of gelatin hydrogels on the migration of gingival epithelial cells was investigated through scratch assay. As shown in Fig. 7b and Fig. S11, compared to the control group and the free Dex group, the gelatin hydrogel groups exhibited an increased migration rate of gingival epithelial cells, suggesting that the gelatin hydrogel itself promoted the wound closure. The MTT assay further demonstrated that these gelatin hydrogels were non-toxic to gingival epithelial cells but also promoted their proliferation, as shown in Fig. 7c–e. This pro-proliferative effect of gelatin is not an isolated observation; previous studies by researchers such as He and Munding et al. [28,53] have reported that gelatin facilitates cell growth through two primary mechanisms: (1) it provides adhesion sites for cell attachment, offering spatial support at the microscale for anchorage-dependent growth; and (2) upon proteolytic hydrolysis, gelatin is broken down into bioactive peptides and amino acids that directly supply nutrients to cells. This explains why the gelatin hydrogel alone promoted ulcer healing in Fig. 5. Given these advantageous properties, we propose that gelatin serves as a more effective drug-loading matrix for oral ulcer treatment compared to widely used but non-degradable polymers in humans, such as chitosan and cellulose.

For the PDA-Dex-PAN loaded gelatin hydrogel used in this study, both gelatin and polylactic acid are approved by the U.S. Food and Drug Administration (FDA) as safe pharmaceutical and food-grade polymers. The only component with potential toxicity is the cross-linker metabolite, 1,3-oxathiolan-2-one. To address this issue, we analyzed 1,3-oxathiolan-2-one in mouse serum using gas chromatography–mass spectrometry (GC–MS). Fig. S12a illustrates the degradation process of the cross-linked gelatin hydrogel under physiological conditions. One day after administration to oral ulcers, the cross-linker metabolite was detectable in the serum (Fig. S12b–l). This metabolite was completely cleared within eight days, indicating that the small molecule 1,3-oxathiolan-2-one did not accumulate long-term in rats. Furthermore, in vitro and in vivo experiments using a high dose of 1,3-oxathiolan-2-one over an eight-day period demonstrated that 1,3-oxathiolan-2-one caused no damage to any rat organs and exhibited no cytotoxicity, as shown in Fig. S13. These results further confirm that the newly developed Dex formulation possesses high safety and holds great promise for the treatment of oral ulcers.

## 4 Conclusion

Enhancing drug delivery efficiency and therapeutic efficacy for oral diseases remains a significant challenge. To address this, we developed an ultra-adhesive gelatin hydrogel by using a symmetric bisuccinimidyl-ester as the cross-linker. This hydrogel demonstrates robust adhesion to skin and mucosal surfaces, resisting erosion by saliva and mechanical shear forces, and can remain in place for several days, making it an excellent drug carrier for oral administration. To further improve delivery efficiency, drugs were encapsulated into polylactic acid nanoparticles (PANs). Through optimized surface modifications, the targeting efficiency of these nanoparticles was enhanced. Using an oral ulcer treatment model, drug-loaded PANs were incorporated into the gelatin hydrogel, which was then adhered to the ulcer site. This approach enabled the sustained release and targeted delivery of dexamethasone (Dex) nanoformulations. In vivo studies confirmed that the Dex-PAN loaded gelatin hydrogel drug delivery system promotes rapid healing of oral ulcers following a single administration. Furthermore, in vitro experiments demonstrated that the gelatin hydrogel is not only non-toxic to gingival epithelial cells but also promotes their proliferation, thereby facilitating mucosal repair. However, the cross-linker used in this study is unstable in solution at room temperature (25 °C) and must be prepared immediately before use. Alternatively, low-temperature storage (e.g., in a refrigerator) substantially enhances the stability of the cross-linker, thus facilitating its clinical application by reducing operational complexity.

## CRediT authorship contribution statement

**Qianwei Tang:** Investigation, Writing – original draft. **Yanlin Chen:** Investigation, Writing – original draft. **Meng Liu:** Investigation. **Xueping Ning:** Data curation. **Quanzhi Chen:** Data curation. **Shengbin He:** Conceptualization, Funding acquisition, Project administration, Supervision, Writing – review & editing.

## Declaration of competing interest

The authors declare that they have no known competing financial interests or personal relationships that could have appeared to influence the work reported in this paper.

## Data Availability

Data will be made available on request.
