# Supplementary material for: Enhancing the therapeutic efficacy of dexamethasone for oral ulcers through adhesive gelatin hydrogel-based nano-delivery system
Source: Mater Today Bio. 2026 Jun 10;39:103341. doi: 10.1016/j.mtbio.2026.103341 (PMC13267620; doi:10.1016/j.mtbio.2026.103341)
Supplement: Multimedia component 1 [file mmc1.docx]

**Supporting Information for**

**Enhancing the therapeutic efficacy of dexamethasone for oral ulcers through adhesive gelatin hydrogel-based nano-delivery system**

Qianwei Tang^1,2,3†^, Yanlin Chen^1,2†^, Meng Liu^1,2†^, Xueping Ning^1,2^, Quanzhi Chen^1,2^, Shengbin He^1,2*^

^1^, Guangxi Medical University, Nanning, Guangxi 530021, P. R. China.

^2^, University Engineering Research Center of Advanced Technologies in Medical and Biological Intelligent Manufacturing, Guangxi Colleges and Universities Key Laboratory of Biological Molecular Medicine Research, School of Basic Medical Sciences, Guangxi Medical University, Nanning, Guangxi 530021, P. R. China.

^3^, Guangxi Medical University College of Stomatology, Guangxi Medical University, Nanning, Guangxi 530021, P. R. China.

*, To whom correspondence should be addressed. E-mail: [comhsb@163.com](mailto:comhsb@163.com) (S. He)

^†^, These authors contributed equally to this work.

This Supporting Information includes Supplementary Protocol 1, Figure S1, Figure S2, Figure S3, Figure S4, Figure S5, Figure S6, Figure S7, Figure S8, Figure S9, Figure S10, Figure S11, Figure S12, and Figure S13.

**Protocol S1**

Firstly, mix 10 mL phosgene (15 % in toluene) with 20 mL 2-hydroxyethyl disulphide (0.2 M in tetrahydrofuran) in a flask. The mixture was stirred for 10 h at room temperature. The solvents were removed through rotary evaporation. After that, 20 mL *N*-hydroxysuccinimide (0.08 g/mL in tetrahydrofuran) and 1000 μL triethylamine were added to the flask. The mixture was incubated at 40 ◦C to react for 18 h. The solvent was removed through rotary evaporation to obtain crude product, which was further purified by silica gel chromatography and recrystallized with icy hexane. Nuclear magnetic resonance spectroscopy (NMR) analysis was carried out to confirm the chemical structure of the product.


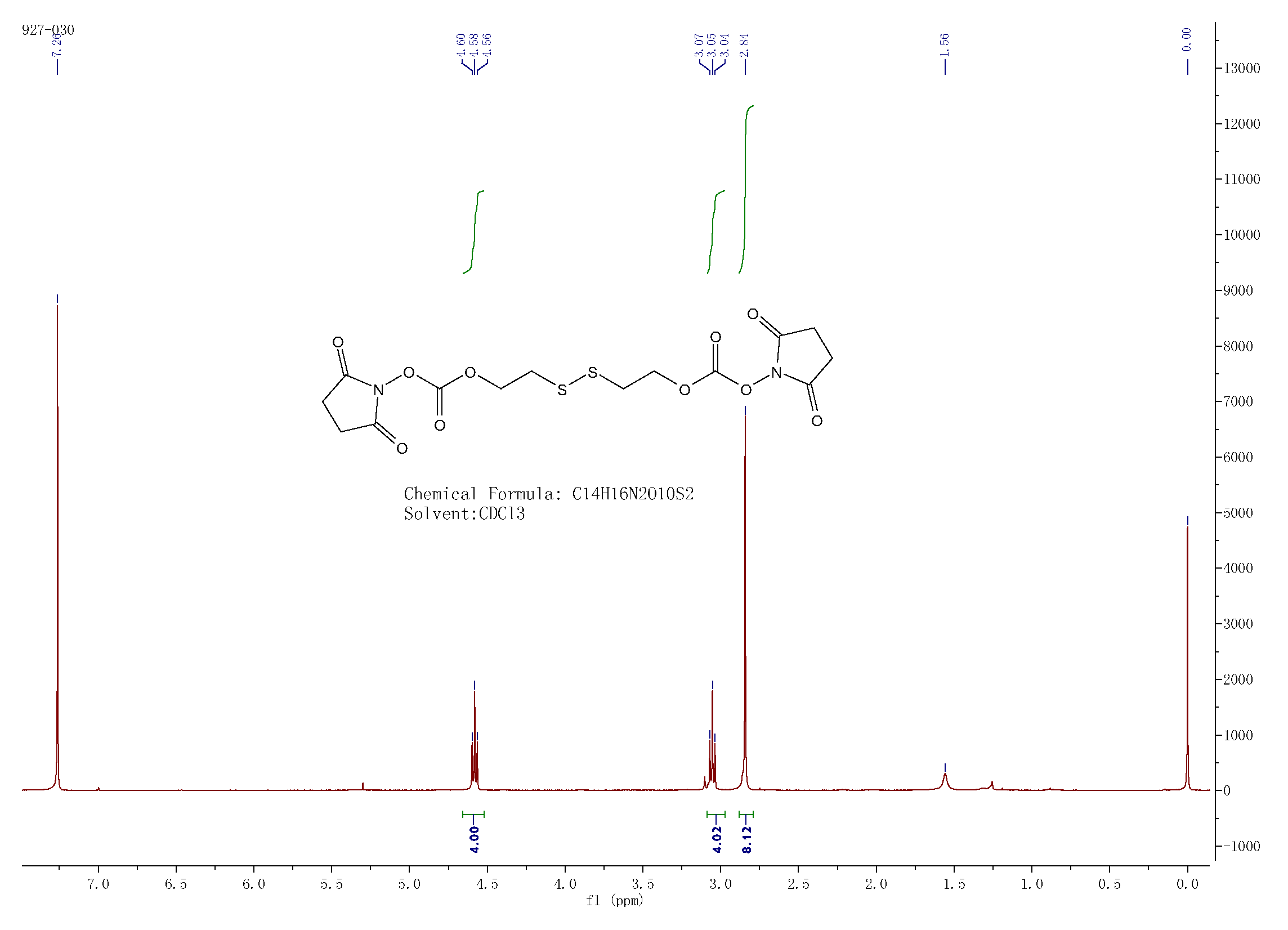


**Figure S1.** LC-MS analysis of the cross-linker


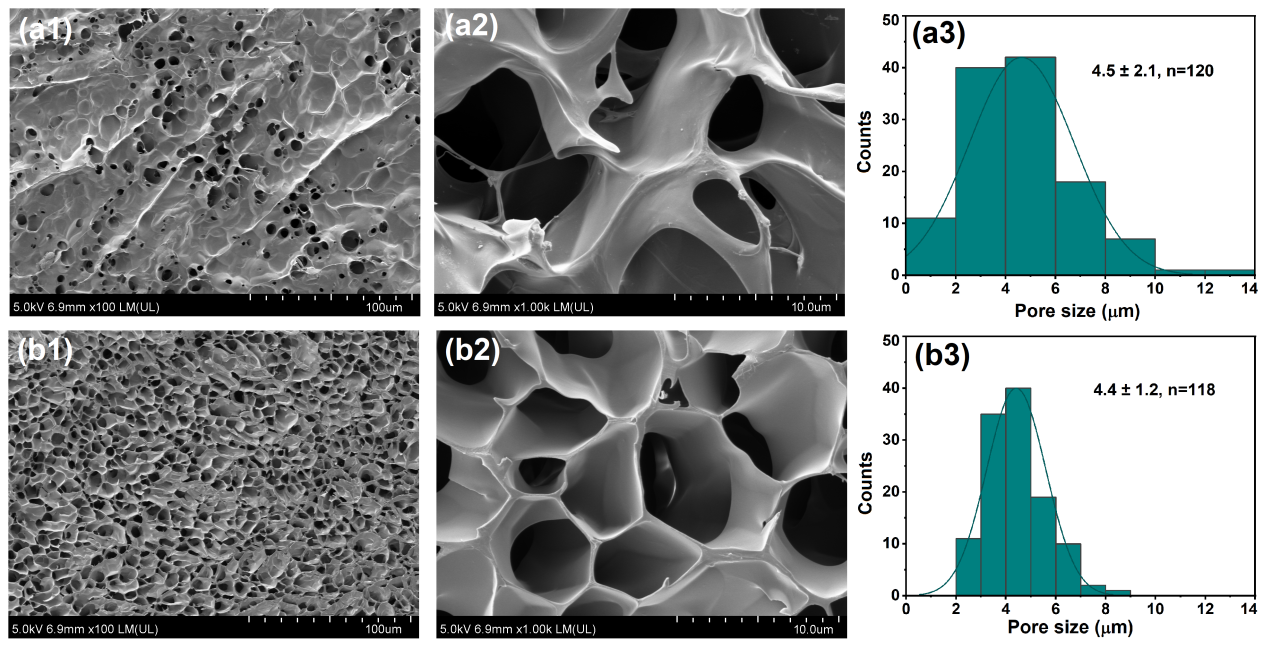


**Figure S2.** Scanning electron microscopy (SEM) observation of the cross-linked gelatin hydrogels (with a cross-linker concentration of 10 mg/mL). (a1) Low-magnification observation of the hydrogel surface morphology. (a2) High-magnification observation of the hydrogel surface morphology. (a3) Statistical distribution of pore sizes on the gel surface (statistical analysis of SEM images by ImageJ software). (b1) Low-magnification observation of the cross-sectional morphology of the hydrogel. (b2) High-magnification observation of the cross-sectional morphology of the hydrogel. (b3) Statistical distribution of pore sizes in the gel cross-section.


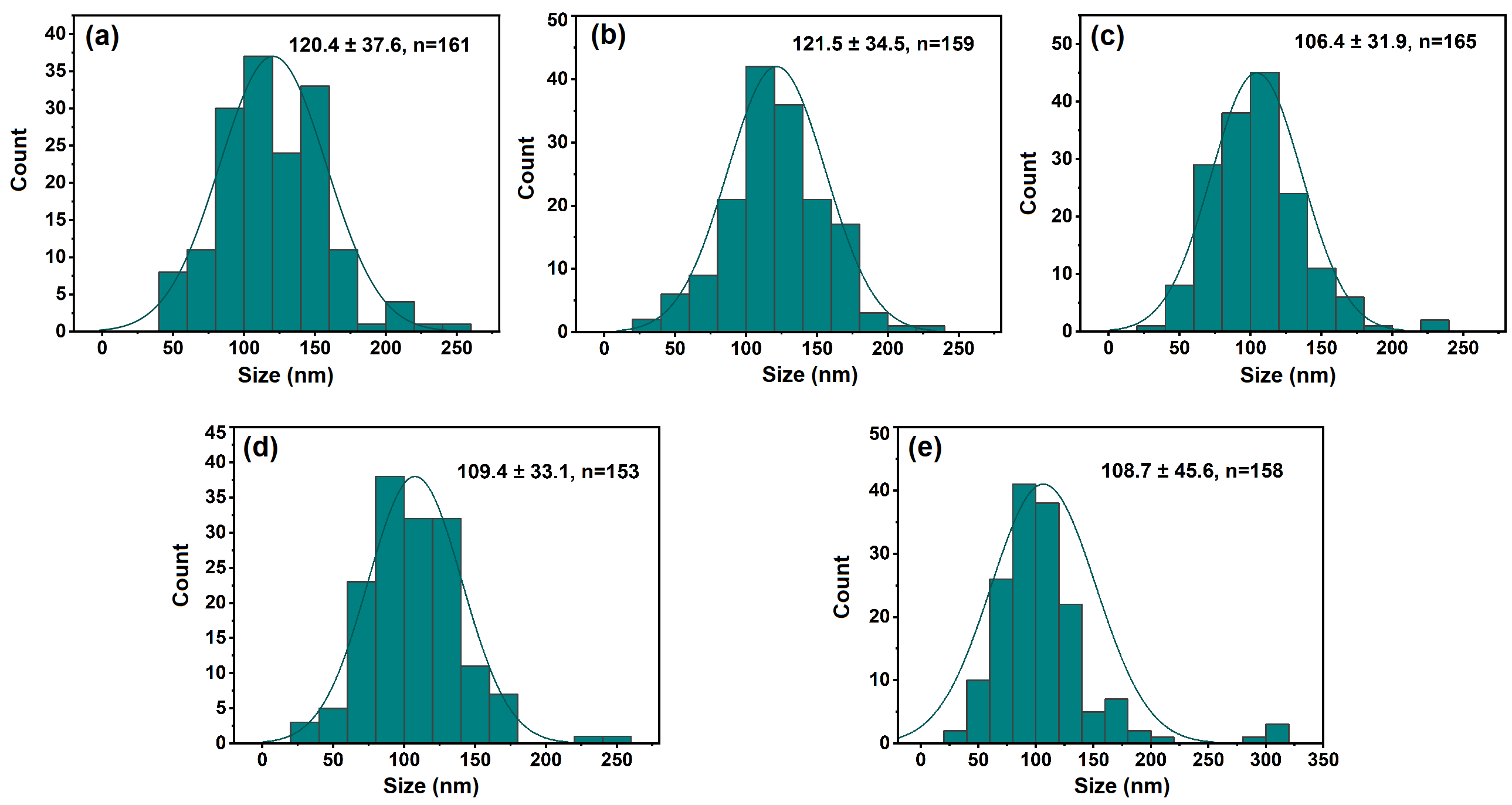


**Figure S3.** Size distribution profiles (statistical analysis of SEM images by ImageJ software) of differently modified polylactic acid nanoparticles. (a) Polylactic acid nanoparticle (PAN). (b) PAN ‌functionalized with dopamine‌ (PDA-PAN). (c) PAN ‌functionalized with polyethylene glycol (PEG-PAN). (d) PAN ‌functionalized with poly-L-lysine (PLL-PAN). (e) PAN ‌loaded with rhodamine B (RhB-PAN).


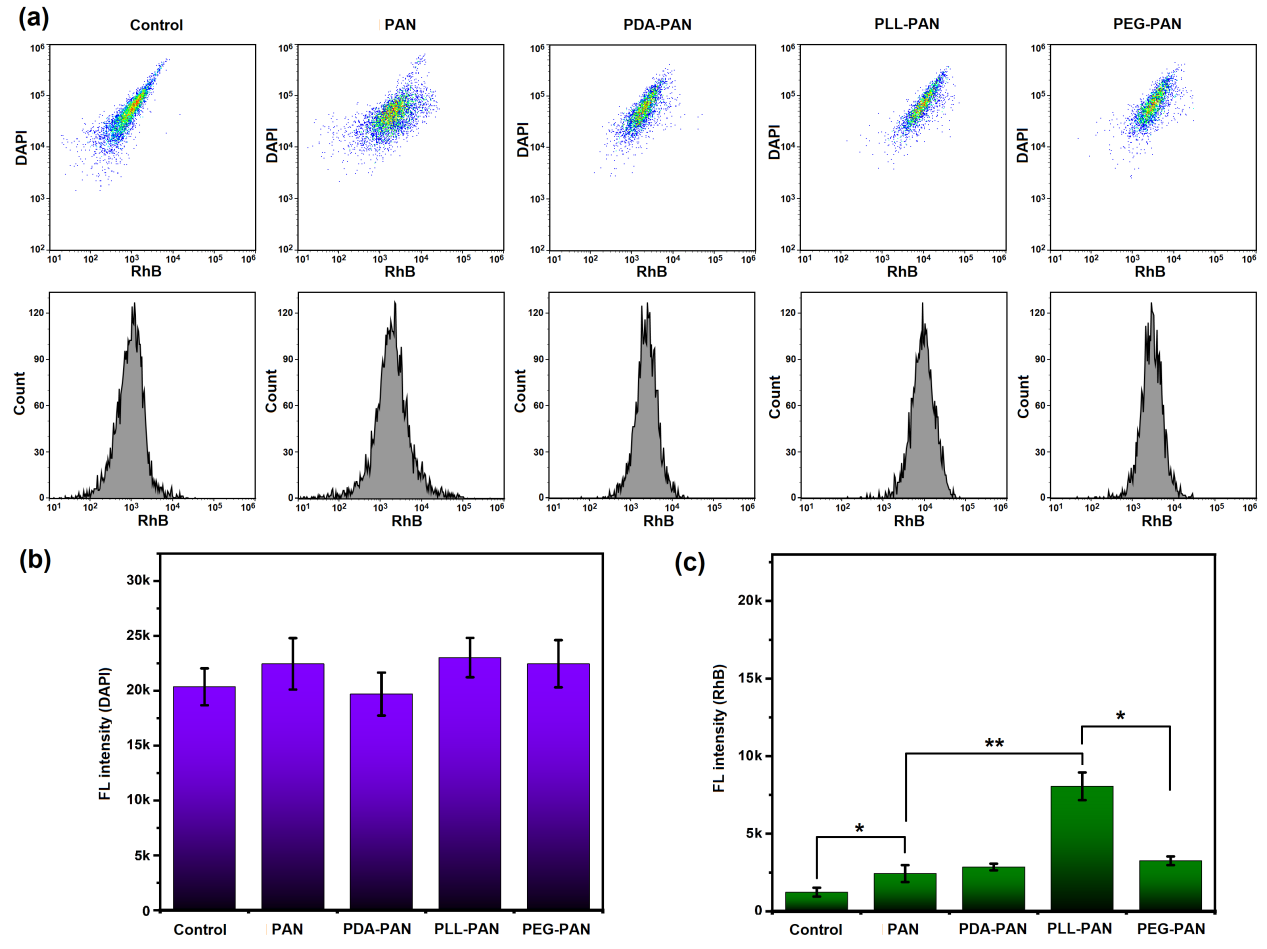


**Figure S4.** Uptake efficiency of differently modified polylactic acid nanoparticles by gingival epithelial cells. (a) Flow cytometry analysis of the nanoparticle uptake. (b-c) Statistical comparison of the corresponding mean fluorescence intensities of DAPI (b) and RhB (c) from FCM analysis. The data are represented as mean ± SD (n = 3). The difference analyses were performed using the Student’s t-test, and the significance was indicated by one asterisk for p < 0.05 and two asterisks for p < 0.01.


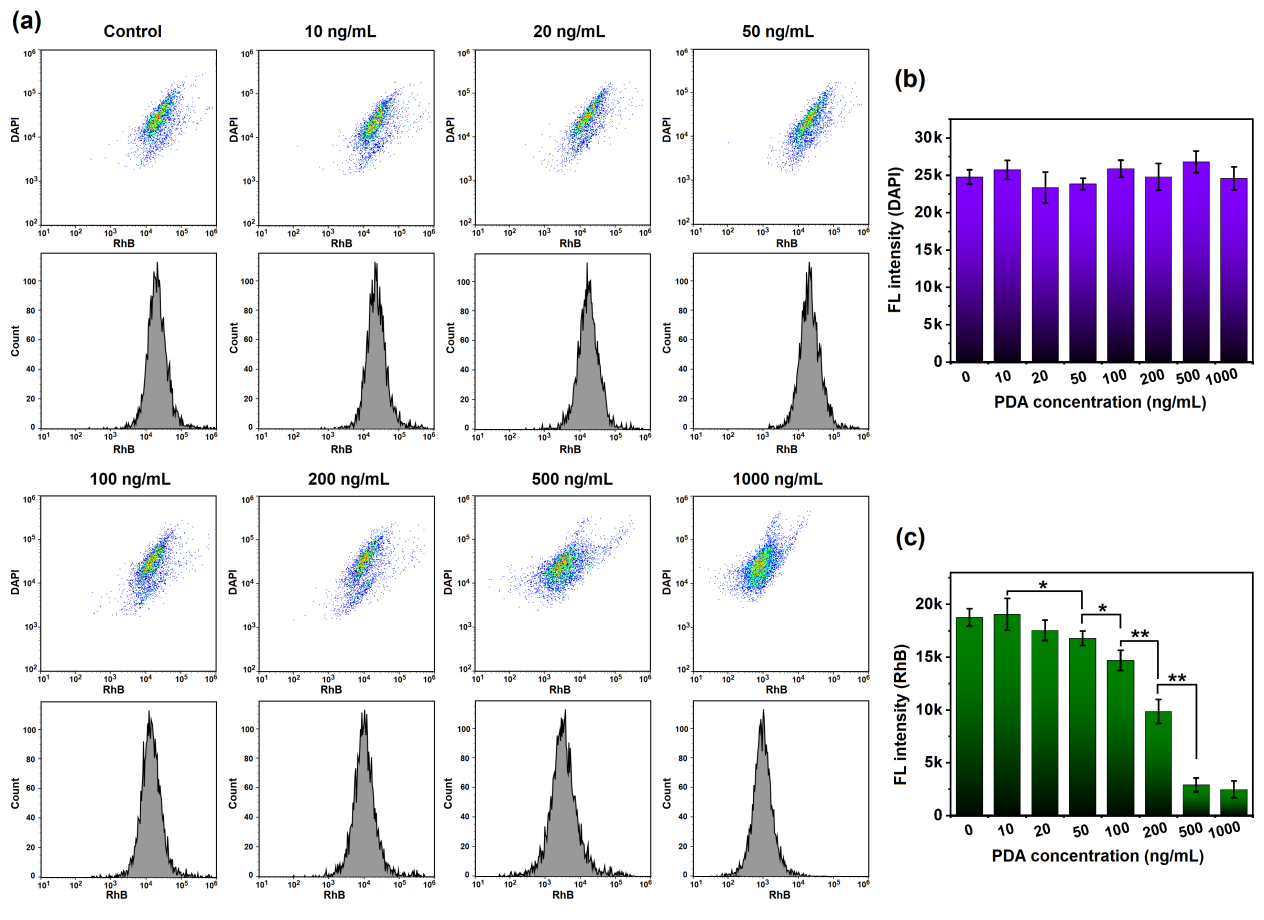


**Figure S5.** Effect of free PDA on the uptake efficiency of PDA-PANs by macrophages. (a) Flow cytometry analysis of the nanoparticle uptake in the presence of free PDA, ranging from 10 to 1000 ng/mL. For control group, no PDA was added. (b-c) Statistical comparison of the corresponding mean fluorescence intensities of DAPI (b) and RhB (c) from FCM analysis. The data are represented as mean ± SD (n = 3). The difference analyses were performed using the Student’s t-test, and the significance was indicated by one asterisk for p < 0.05 and two asterisks for p < 0.01.


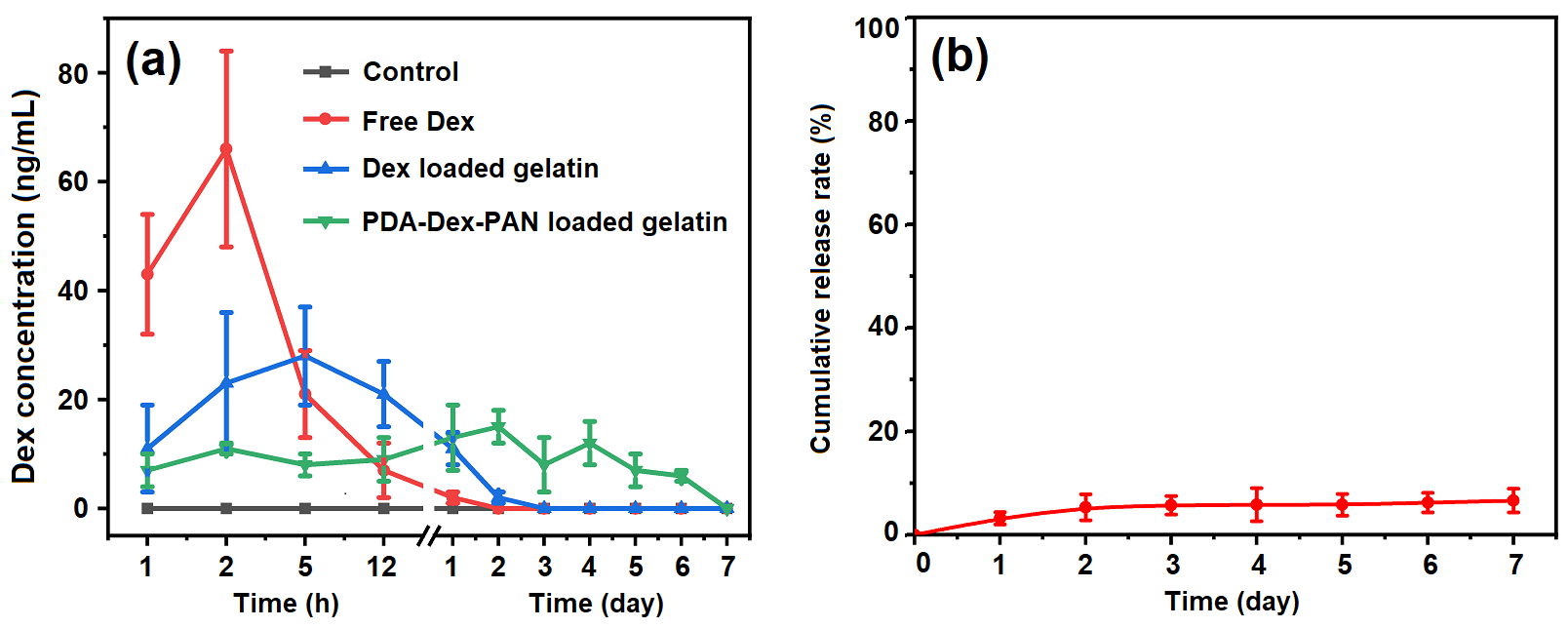


**Figure S6.** Release dynamic of Dex. (a) Variation in serum Dex concentration as a function of time after application of different formulations via the buccal. For control, no Dex was applied. For free Dex group, free Dex was directly applied to the buccal. Serum samples were analyzed by High Performance Liquid Chromatography (HPLC) as described by Sun et al.[1]. The data are represented as mean ± SD (n = 6). (b) Release of Dex from PDA-Dex-PAN into artificial serum. The data are represented as mean ± SD (3 = 3).


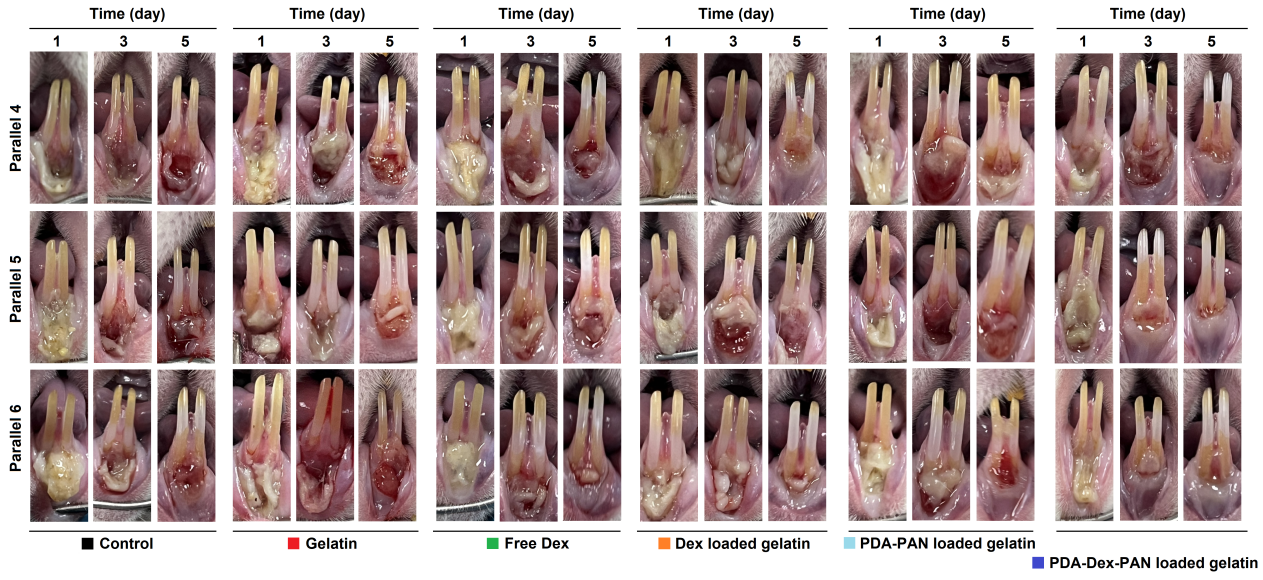


**Figure S7.** Therapeutic effects of different dexamethasone formulations on rat oral ulcers.


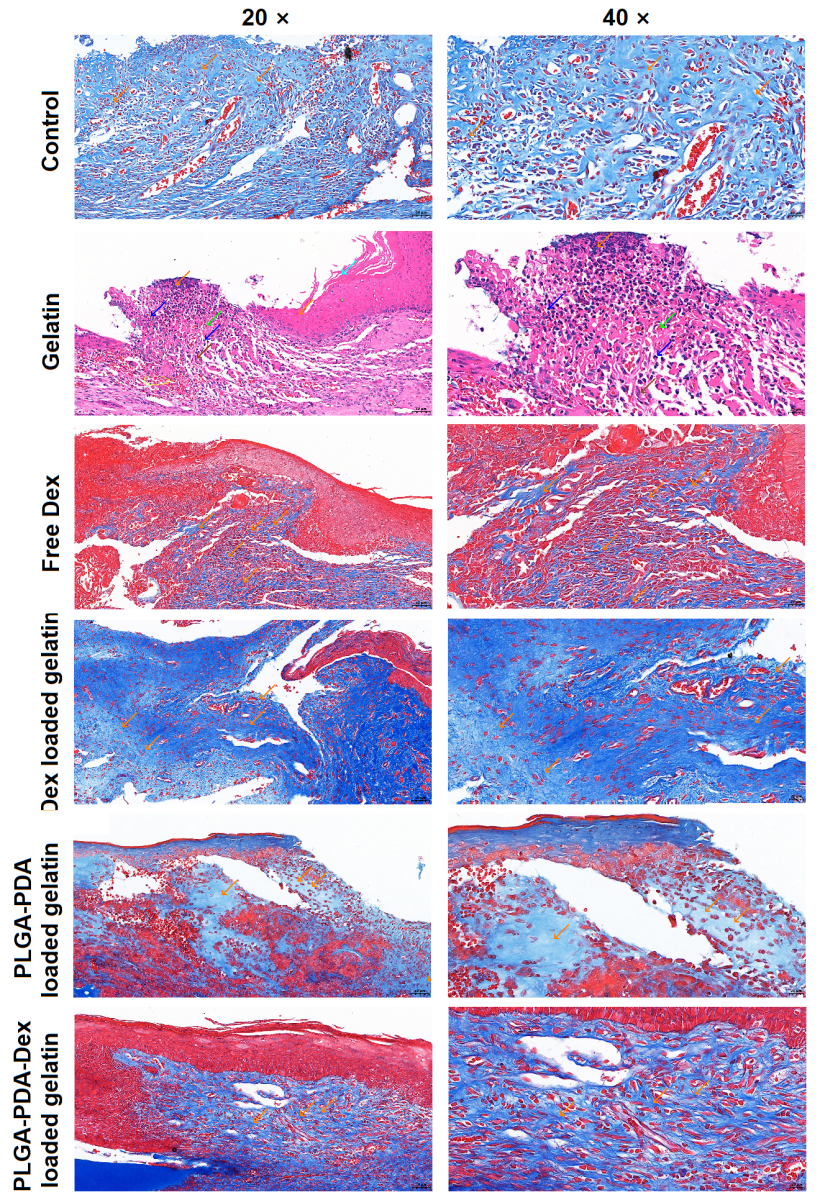


**Figure S8.** Masson staining observation of the ulcer sites in rat oral mucosa after treatment with different dexamethasone formulations. The orange arrow indicates visible gingival tissue with collagen fiber proliferation, characterized by fine fibers and irregular arrangement.


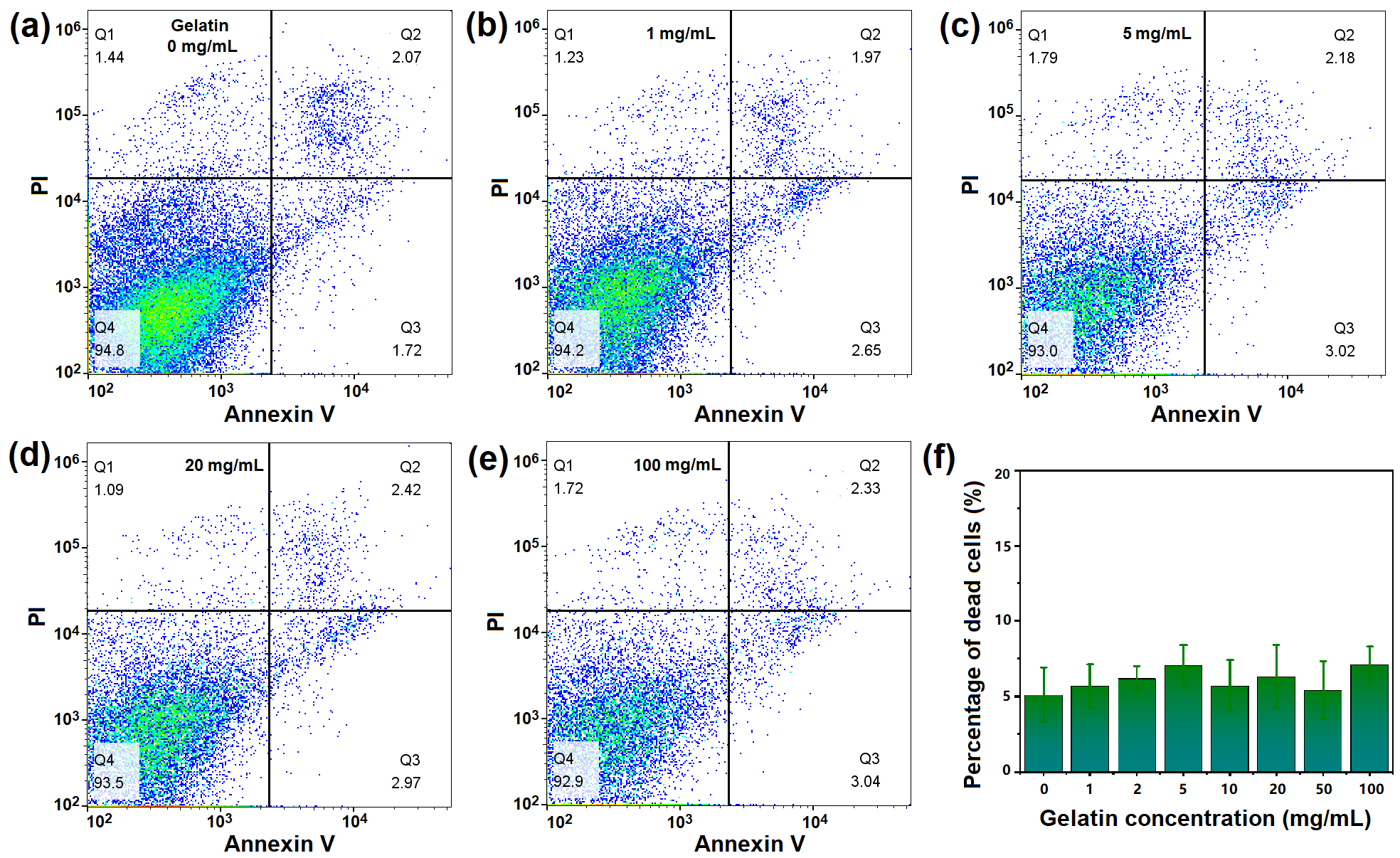


**Figure S9.** Apoptosis evaluation of the gingival epithelial cells grown in the presence of gelatin of different concentrations. (a-g) Representative scatter plots for cells, with gelatin concentrations of 0 mg/mL for (a),1 mg/mL for (b), 5 mg/mL for (c), 20 μg/mL for (d), and 100 μg/mL for (e). (f) Comparison in dead cell numbers between different gelatin concentrations. The data are represented as mean ± SD (n = 3).


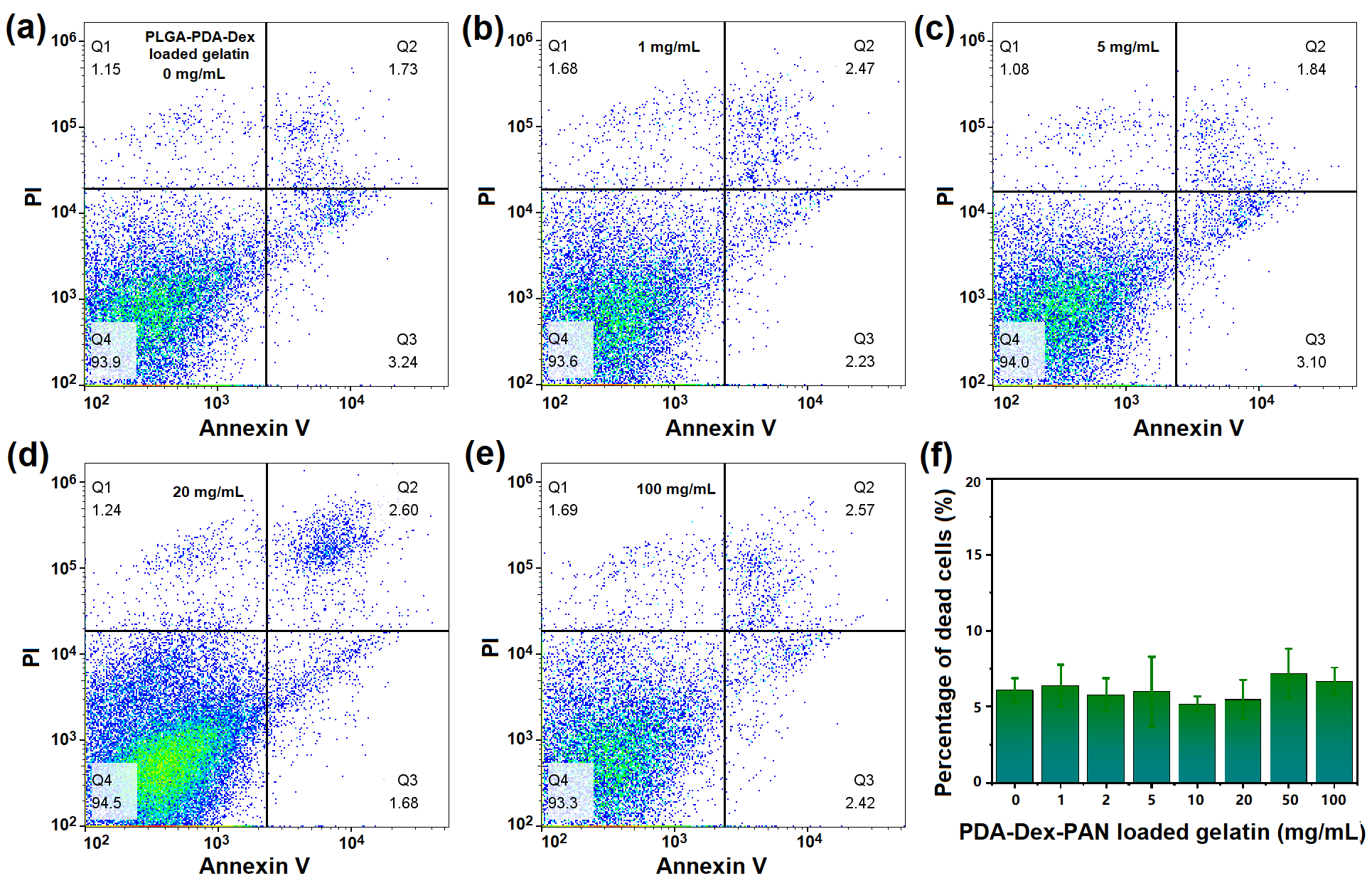


**Figure S10.** Apoptosis evaluation of the gingival epithelial cells grown in the presence of PDA-Dex-PAN loaded gelatin of different concentrations. (a-g) Representative scatter plots for cells, with PDA-Dex-PAN loaded gelatin concentrations of 0 mg/mL for (a),1 mg/mL for (b), 5 mg/mL for (c), 20 μg/mL for (d), and 100 μg/mL for (e). (f) Comparison in dead cell numbers between different PDA-Dex-PAN loaded gelatin concentrations. The data are represented as mean ± SD (n = 3).


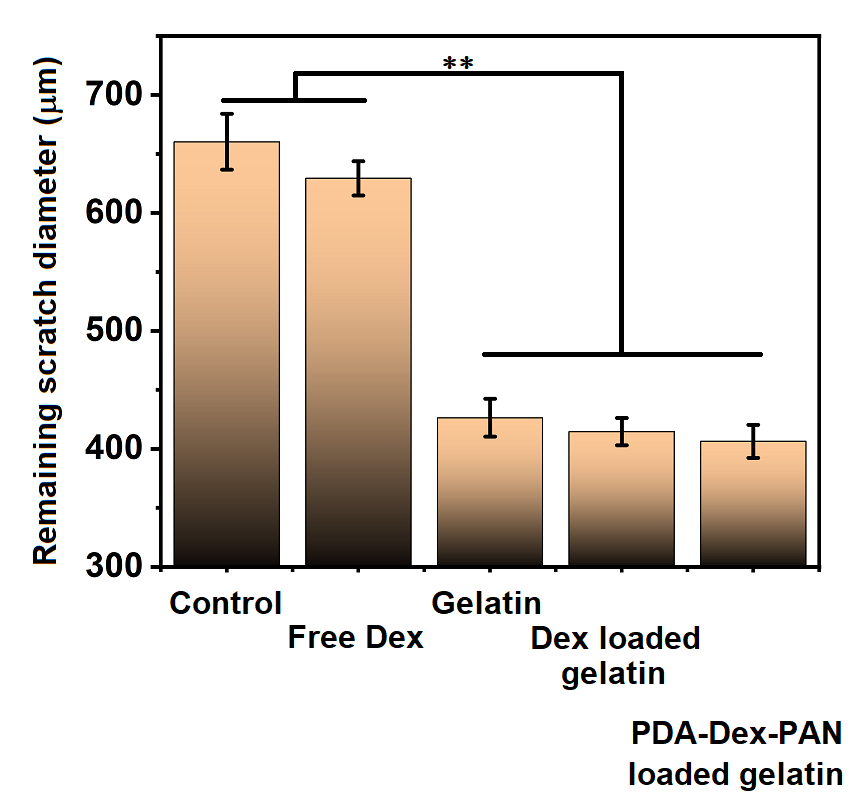


**Figure S11.** Remaining scratch diameter for the scratch assay. The data are represented as mean ± SD (n = 3). The difference analyses were performed using the Student’s t-test, and the significance was indicated by two asterisks for p < 0.01.


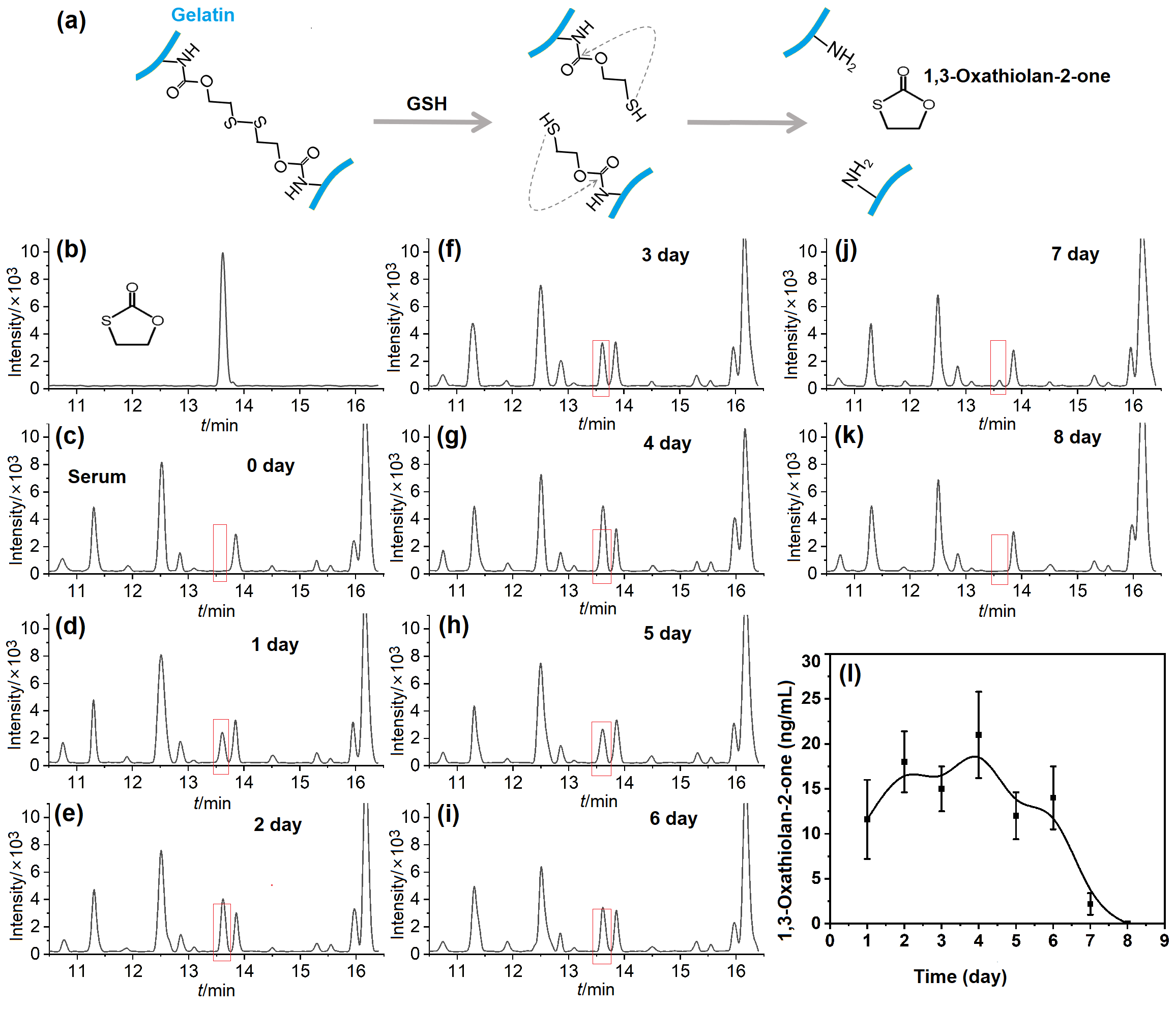


****Figure S12.** Serum metabolite analysis of the cross-linked gelatin hydrogels.** (a) Schematic illustration of the degradation of the cross-linked gelatin material. The cross-linker is reduced by glutathione (GSH) in the physiological environment and undergoes a self-elimination reaction [2,3] to generate the metabolite 1,3-oxathiolan-2-one. (b) Standard of 1,3-oxathiolan-2-one (50 ng/mL). (c–k) Analysis of serum metabolites from rats after application of the cross-linked gelatin hydrogel to oral ulcer sites for **various time points (from day 0 to day 8)**. (l) Serum pharmacokinetic profile derived from six replicate experiments. The data are represented as mean ± SD (n = 6). Serum samples were analyzed by gas chromatography–mass spectrometry (GC–MS) as described by Colldén et al.[4]


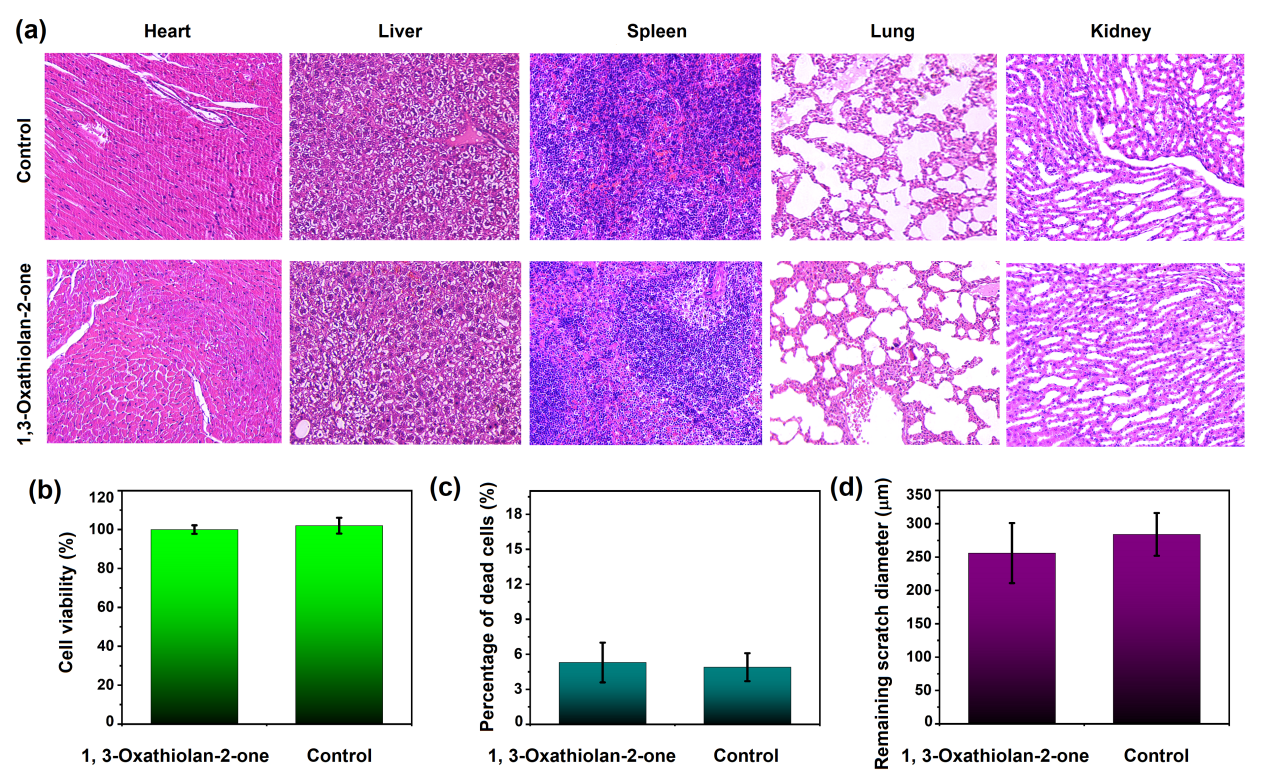


****Figure S13.** Long-term toxicity for high-dose administration of 1,3-Oxathiolan-one. (a) H&E staining of various rat organs following administration of 1,3-Oxathiolan-one.** In the experimental group, 1,3-Oxathiolan-one was administered intravenously at a dose of 100 ng/g body weight once daily for 8 consecutive days. The control group received physiological saline instead. **(b–d) Effects of 1,3-Oxathiolan-one on the proliferation (b), apoptosis (c), and migration (d) of gingival epithelial cells.** In the experimental group, cells were cultured with 1,3-Oxathiolan-one at a concentration of 100 ng/mL, with the medium (containing 1,3-Oxathiolan-one) replaced daily for 8 consecutive days. The data are represented as mean ± SD (n = 3).

Reference:

1. Sun J, Ning X, Cui L., Ling M., He S, (2020) Assembly of "carrier free" enzymatic nano-reporters for improved elisa. The Analyst 145:6541-6548*.*
2. Riber CF, Smith AAA, Zelikin AN, (2015) Self-immolative linkers literally bridge disulfide chemistry and the realm of thiol-free drugs. Adv. Healthcare Mater. 4:1887–1890.
3. Jones LR, Goun EA, Shinde R, Rothbard JB, Contag CH, Wender PA, (2006) Releasable luciferin-transporter conjugates: tools for the real-time analysis of cellular uptake and release. J. Am. Chem. Soc. 128:6526-6527.
4. Colldén H, Nilsson ME, Norlén AK, Landin A, Windahl SH, Wu JY, Gustafsson KL, Poutanen M, Ryberg H, Vandenput L, Ohlsson C, (2022) Comprehensive sex steroid profiling in multiple tissues reveals novel insights in sex steroid distribution in male mice. 163(3):bqac001.
